# Crystal Facet‐Polysaccharide Matching in CFS‐P Nanocrystals Drives Fungal Uptake, Vacuole Destruction, and Selective Antifungal Activity

**DOI:** 10.1002/advs.202524219

**Published:** 2026-04-20

**Authors:** Zhaohui Wang, Xiaojin Deng, Xujuan Huang, Yuhang Pan, Yao Qin, Lu Zhang, Le Xu, Tianyi Qin, Deteng Zhang, Yi Ma, Yalong Wang

**Affiliations:** ^1^ State Key Laboratory of Digital Medical Engineering School of Biomedical Engineering Sanya Research Institute of Hainan University Hainan University Sanya China; ^2^ Department of Biomedical Engineering School of Engineering China Pharmaceutical University Nanjing China; ^3^ Khoury College of Computer Sciences Northeastern University Boston Massachusetts USA; ^4^ Institute of Neuroregeneration and Neurorehabilitation Qingdao University Qingdao China

**Keywords:** antifungal, facet‐engineered, mannan binding, nanocrystals, organelle‐targeting, vacuole disruption

## Abstract

The lack of selective antifungal agents remains a major clinical challenge. Here, we discover facet‐polysaccharide matching between polyvinylpyrrolidone‐directed tetragonal CuFeSe_2_ nanocrystals (CFS‐P) and fungi drives ultrapotent and highly selective fungicidal activity. High‐index facets uniquely exposed on CFS‐P show markedly enhanced affinity for mannan, the dominant polysaccharide of the fungal outer cell wall, as validated by DFT, molecular dynamics, and calorimetry. Mannan recognition promotes efficient fungal internalization of CFS‐P and its preferential accumulation in vacuoles, leading to vacuolar cavitation, calcium overload, mitochondrial hyperpolarization, and transcriptional reprogramming of metal and vacuole‐associated pathways. CFS‐P achieves therapeutic efficacy comparable to amphotericin B while exhibiting superior mammalian biocompatibility. This work reveals a previously unrecognized wall‐vacuole disruptive antifungal mechanism and enlightens the exploration of facet‐encoded biological recognition in anti‐infective design.

## Graphical abstract

Facet‐engineered PVP‐capped CuFeSe_2_ nanocrystals exhibit potent, selective antifungal activity by strongly binding cell‐wall mannan, enabling vacuolar entry and organelle disruption. High‐index facets show superior mannan affinity, validated by theoretical calculations and experiments. CFS‐P induces vacuolar cavitation, mitochondrial abnormalities, and robust fungal killing with excellent efficacy and biocompatibility, establishing a paradigm for nanocrystal targeting cell walls and organelles.

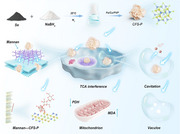

## 1 Introduction

Current clinical antifungal therapy is limited by the increasing emergence of drug resistance, a scarcity of drug categories, and severe side effects. A major hurdle in antifungal drug design is inherent toxicity, as fungi and mammalian cells are both eukaryotes and share common cellular structures and biochemical pathways [1]. For instance, polyene drugs such as amphotericin B disrupt membrane integrity by binding to ergosterol but also interact with cholesterol in human cell membranes, leading to red blood cell hemolysis and renal tubular epithelial damage [2, 3, 4]. Drugs targeting unique fungal cellular structures are promising to exhibit low toxicity. To date, only echinocandins, which inhibit glucan synthase to disrupt cell wall formation, serve as a last‐line antifungal class with satisfactory biocompatibility [5, 6, 7, 8]. However, mutations in the genes regulating this enzyme have led to acquired resistance in *Candida* species [9, 10]. Since the introduction of echinocandins in the 2000s, no new antifungal drugs targeting fungal organelles have been launched [11, 12].

The most distinct structural differences between fungi and mammalian cells are the cell wall and the vacuole. Essential for fungal survival, the cell wall is a conserved structure composed of an inner layer of β‐glucan and an outer layer of N‐manna [13, 14]. The outer wall provides adhesive, shielding, and immunological functions, while the inner wall forms a viscoelastic framework that defines morphology and strength [15]. These characteristics make the cell wall an attractive target for drug development, yet few cell wall‐targeting agents besides echinocandins have been successfully marketed [16]. Fungal vacuoles share functional similarities with mammalian lysosomes but represent a unique organelle in fungi, involved in cargo degradation, ion homeostasis, and stress tolerance [17, 18]. Recent studies report that disrupting vacuolar function impairs the pathogenicity of *C. albicans* by interfering with hyphal penetration [19, 20]. Furthermore, vacuolar uptake of drugs is linked to echinocandin resistance [21]. Given the exclusivity of the cell wall and vacuole in fungi, the discovery of a dual organelle‐disrupting agent with excellent biocompatibility is highly desirable.

In the search for novel fungicidal substances, agents at the nano or supramolecular scale have emerged as promising alternatives [22]. Many of these structures serve as drug carriers for efficient delivery of existing antifungals or as platforms for multimodal therapy, leveraging optical properties [23, 24]. However, their multi‐component and complex nature often hinders clinical translation. Moreover, optical treatments using nano‐formulations are generally limited to superficial fungal infections and are less effective against deep‐tissue invasive infections such as candidemia [25, 26]. The reported nano‐agents with direct fungicidal activity mainly rely on chemical catalytic properties, for example, nanozymes with peroxidase‐like activity that catalyze free radical generation from exogenous H_2_O_2_ in infected areas [27, 28], which are highly dependent on specific chemical environments [29].

Nano‐crystals with potent intrinsic antifungal properties that specifically target fungi remain underexplored, particularly those capable of disrupting fungi‐specific organelles while sparing mammalian cells. The crystal facet, referring to the crystallographic atomic arrangement at the surface, is a key factor determining the intrinsic properties of crystalline nanoparticles, including catalytic activity, electronic behavior, and molecular affinity [30, 34]. Controlling crystal growth profoundly influences crystal morphology, which follows a kinetic process under colloidal conditions [35]. Typically, facets with high surface energies grow rapidly and vanish quickly, resulting in the exposure of low‐energy surfaces. Crystal facet engineering has been applied in photocatalysis [36], solar cell development [37], and electrochemical reactions [38]. However, interactions between crystal facets and biological molecules are far less studied. To the best of our knowledge, the influence of crystal facets on antifungal activity has not been previously reported.

**FIGURE 1:**
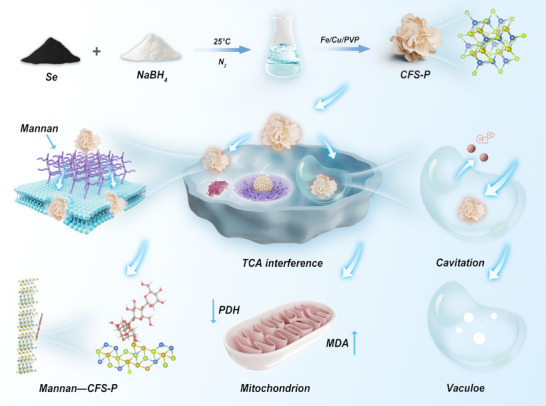
Scheme of the synthesis and fungicidal mechanism of CFS‐P. CFS‐P is synthesized by aqueous precipitation with PVP as a capping ligand at room temperature. Upon incubation with fungal cells, the CFS‐P adheres to the fungal surface by strong affinity of specific facets to mannan, the major component of the fungal cell wall. This interaction facilitates efficient fungal uptake and vacuoles internalization, leading to vacuolar cavitation, mitochondrion damage, and ultimately fungal death.

In this study, we unveil a novel antifungal mechanism of a specific form of copper–iron–selenium nano‐crystal, CFS‐P, building on prior findings from our group [39]. It is demonstrated that the high‐index facets of semiconductor CuFeSe_2_ nanocrystals exhibit strong affinity for mannan, the major component of the fungal outer cell wall. This facet–mannan interaction promotes efficient crystal internalization into vacuoles, resulting in internal cavitation and subsequent vacuolar damage (Figure 1). Importantly, we identify PVP as the key capping agent that reduces the surface energy of high‐index facets, facilitating their exposure. We also validate the therapeutic efficacy and toxicity profile of CFS‐P in vitro and in vivo. This pioneering work may inspire the development of a new class of biocompatible semiconductor antibiotics based on facet engineering targeting fungal organelles.

## 2 Results and Discussion

### 2.1 Fungicidal Activity of Specific CuFeSe_2_ Crystals

**FIGURE 2:**
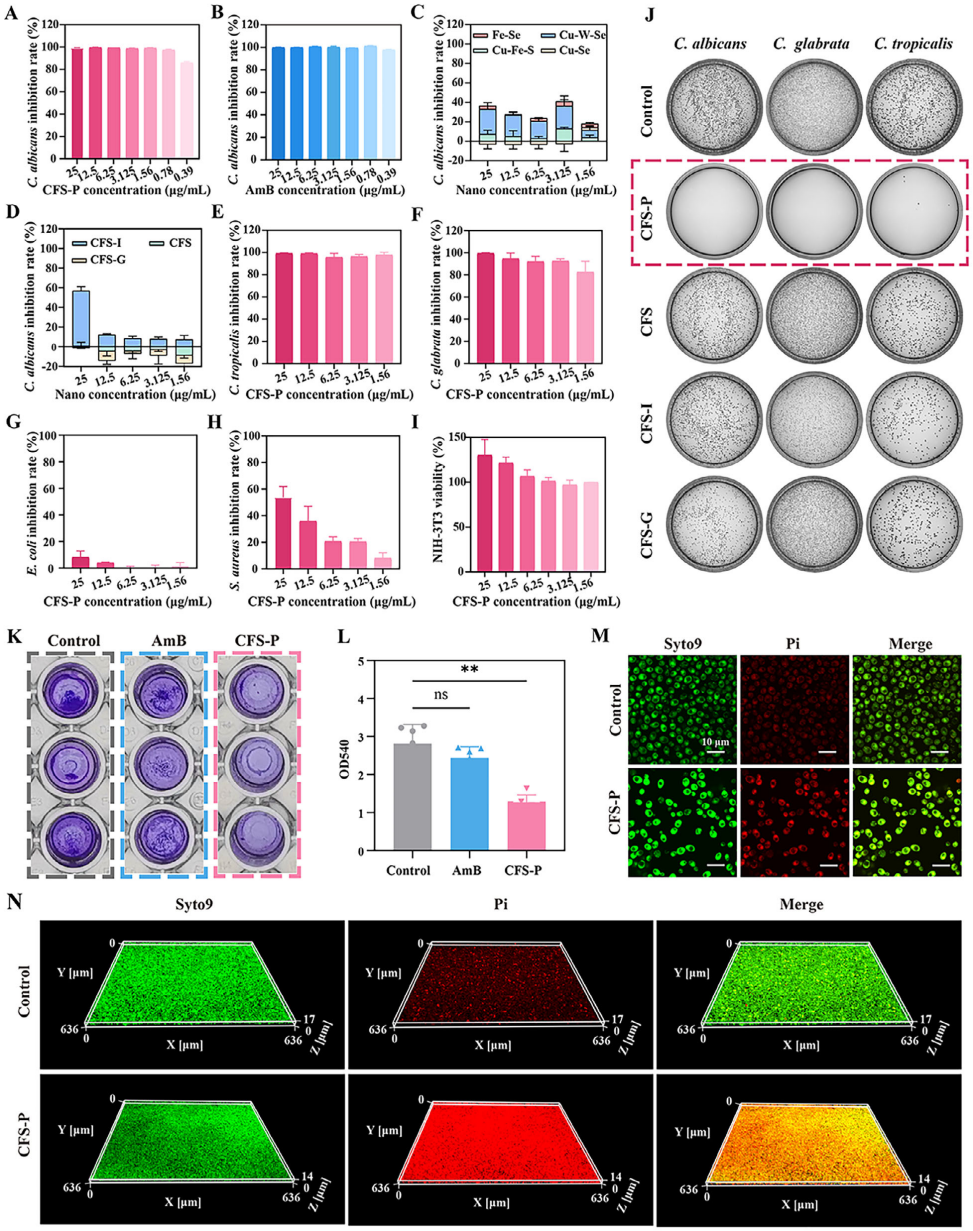
Comparison of fungicidal activity of different crystals. (A) Inhibitory rates of CFS‐P at different concentrations against *C. albicans*. (B) Inhibitory rates of AmB at different concentrations against *C. albicans*. (C) Inhibitory rates of the nanocrystals with different metal combinations against *C. albicans*. (D) Inhibitory rates of CuFeSe_2_ with different capping agents against *C. albicans*. (E–G) Inhibitory rates of CFS‐P at different concentrations against *C. tropicalis* (E), *C. glabrata* (F), *E. coli* (G), and *S. aureus* (H). (I) Viability of NIH‐3T3 cells after incubation with different concentrations of CFS‐P. (J) Plating images of the inhibitory effects of CuFeSe_2_ with different capping agents against *C. albicans*, *C. glabrata*, and *C. tropicalis*. (K, L) Crystal violet staining (K) and the corresponding 540 nm absorbance (L) of biofilms treated with CFS‐P, AmB, and the Control group (Data were presented as mean ± SD, *n* = 6, **p < 0.01 and ns, not significant) (M) SYTO‐9/PI fluorescence staining images after co‐incubation with CFS‐P. (N) *Z*‐axis SYTO‐9/PI fluorescence staining images of biofilms. (For A–J, data were presented as mean ± SD, *n* = 3).

The optimized aqueous precipitation method provides a versatile wet‐chemistry route for the facile synthesis of hydrophilic semiconductor nanocrystals. In our previous work, CuFeSe_2_‐PVP was unexpectedly found to exhibit high fungicidal activity [39], but the underlying mechanism remained unclear. Here, using PVP as the capping agent, we prepared a series of nanocrystals with different elemental compositions to investigate the correlation between metal composition and fungicidal efficacy. The results showed that only CFS‐P effectively inhibited the growth of *C. albicans*, with inhibition rates comparable to those of the first‐line clinical antifungal drug AmB (Figure 2A,B). In contrast, other crystals synthesized by the same method, such as Fe–Se, Cu–Se, Cu–W–Se, and Cu–Fe–S, showed minimal inhibition at concentrations below 25 µg/mL (Figure 2C). These findings indicate that the elemental composition of the nanocrystals plays a vital role in their intrinsic antifungal efficacy and that the combination of Cu, Fe, and Se is essential for antifungal performance. To examine the influence of capping agents with the fixed metal combination, we prepared pure CuFeSe_2_ (CFS) and the variants capped with PEG (CFS‐G) or PEI (CFS‐I), and compared their fungal inhibition effects, where PEG represented for polyethylene glycol and PEI for polyethyleneimine. Intriguingly, none of CFS, CFS‐G, or CFS‐I showed obvious fungicidal activity (Figure 2D). Moreover, CFS‐P also potently inhibited two other common *Candida strains*, *C. tropicalis* and *C. glabrata*, demonstrating a relatively broad antifungal spectrum (Figure 2E,F,J), whereas CFS, CFS‐G, and CFS‐I showed no inhibitory effects (Figure 2J). Notably, CFS‐P exhibited negligible inhibition effect toward bacteria and showed excellent biocompatibility in mammalian cells (Figure 2G–I), suggesting that its specificity is likely associated with unique fungal components. Furthermore, CFS‐P was capable of eradicating fungal biofilms that confer strong resistance to multiple antibiotics. Crystal violet staining provided a quantitative comparison of biofilm eradication between CFS‐P and AmB. Absorbance measurements at 540 nm indicated that CFS‐P significantly reduced fungal biofilm formation, with efficacy superior to that of AmB (Figure 2K,L). Confocal fluorescence imaging of biofilms revealed a pronounced increase in red PI signal in both the floating fungi and the biofilm, indicating extensive fungal cell death following CFS‐P treatment (Figure 2M,N). Measurement of the three‐dimensional imaging showed that biofilm thickness decreased significantly from approximately 17 µm to 14 µm after CFS‐P treatment (Figure 2N). Although CFS‐P and amphotericin B showed comparable inhibitory effects against planktonic fungal cells, fungal biofilms are known to exhibit additional protective mechanisms that disproportionately reduce the efficacy of conventional small‐molecule antifungals, including restricted drug penetration through the extracellular matrix, sequestration of antifungal agents by matrix polysaccharides, and changes in ergosterol composition [40]. The superior antibiofilm activity of CFS‐P is most likely attributable to its ability to avoid being largely blocked by the polysaccharide‐rich biofilm matrix. In addition, to rule out the possibility that the observed antifungal activity is mediated by the leaching of metal ions, the antifungal efficacy of free Cu^2^^+^, Fe^2^^+^, and Fe^3^^+^ were also tested, which did not exhibit observable antifungal activity even at a high concentration of 25 µg/mL, and certain concentrations of the ions even promoted fungal growth to some extent (Figure S1). To clarify whether ROS contributes directly to fungal cell death, we have performed the ROS‐quenching experiment, in which 3 different ROS scavengers were added when CFS‐P was incubated with the fungi. None of these scavengers measurably attenuated the antifungal activity of CFS‐P (Figure S2). This result indicates that ROS generation is unlikely to be the primary upstream driver of fungal killing. Taken together, these results suggest that both the metal combination and the choice of capping agent are critical determinants of the fungicidal properties of materials synthesized via aqueous precipitation, and the activity is dependent on the intrinsic features of the intact CFS‐P nanocrystal system, rather than metal ions leaching or ROS generation.

### 2.2 Characterization of CFS‐P Crystals and Fungal Uptake

**FIGURE 3:**
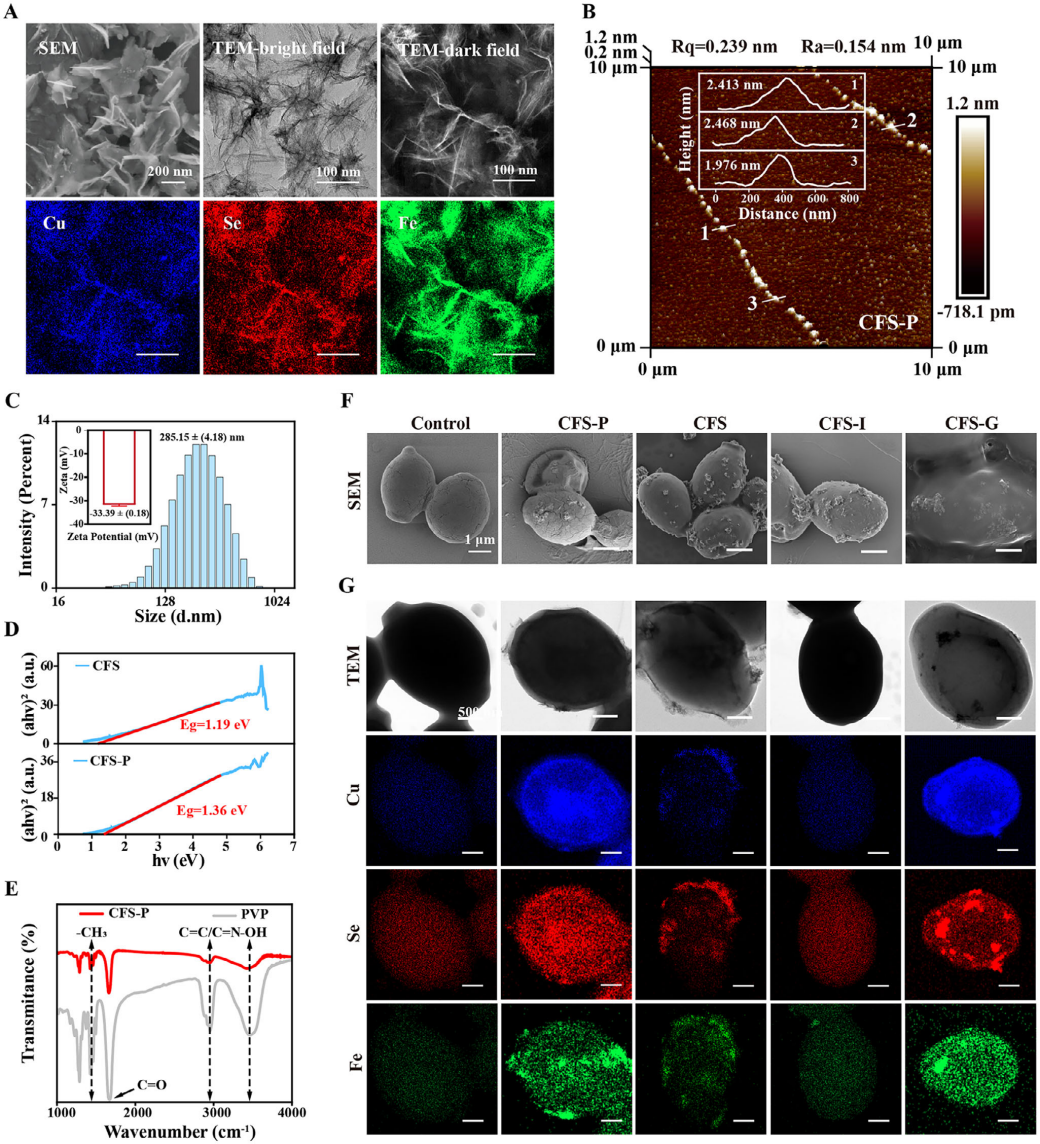
Fungal uptake of different CuFeSe_2_ crystals. (A) Scanning electron microscopy (SEM) and transmission electron microscopy (TEM) images, and elemental mapping (Cu, Fe, Se) of CFS‐P. (B) Atomic force microscopy (AFM) topography image of CFS‐P. (C) Size distribution and zeta potential of CFS‐P. (Data were presented as mean ± SD, *n* = 3) (D) UV–visible diffuse reflectance spectra (UV–vis DRS) of CFS‐P and CFS. (E) Comparative FTIR spectra of CFS‐P and CFS. (E). (F) SEM image of nanomaterial adherence on fungal surface. (G) TEM image and elemental mapping (Cu, Fe, Se) of the fungi treated with CFS‐P.

To understand the marked difference in fungicidal efficacy, we examined the morphological characteristics of the CuFeSe_2_ nanocrystals and visualized their interactions with fungi. Different capping agents resulted in distinct crystal shapes (Figure 3A and Figure S3). Specifically, CFS‐P nanocrystals adopted a linear arrangement, interweaving to form maple leaf‐like sheets, with a mean diameter of 285.15 ± (4.18) nm and a zeta potential of −33.39 ± (0.18) mV (Figure 3A,C). This distinctive morphology was further confirmed by EDS metal element mapping, which showed stronger signals along the linear direction (Figure 3A). Atomic force microscopy (AFM) corroborated the linear particle arrangement and provided measurements of the thickness and roughness of CFS‐P (Figure 3B). Other crystals, including CFS, CFS‐I, and CFS‐G, were considerably thicker than CFS‐P, as indicated by AFM height measurements (Figure S4). These results suggest that PVP promotes one‐dimensional growth of CuFeSe_2_ nanocrystals, implying the exposure of unique facets. Additionally, the bandgap of CFS‐P was larger than that of pristine CFS (Figure 3D). FTIR spectra of CFS‐P showed characteristic peaks similar to those of PVP, confirming the successful capping (Figure 3E). In contrast, Raman signals related to PVP were significantly attenuated in CFS‐P (Figure S5), likely due to restricted vibrational freedom of PVP molecules anchored to the nanocrystal surface, reducing changes in molecular polarizability and thus Raman scattering intensity.

SEM imaging revealed that all nanocrystals adhered effectively to the fungal outer wall, despite their differences in inhibition efficacy (Figure 3F). This suggests that CFS‐P may act intracellularly to disrupt homeostasis, rather than merely through surface attachment and wall disruption. To test this hypothesis, we examined cellular uptake of the four nanocrystals using TEM and elemental mapping (Figure 3G). As expected, only CFS‐P efficiently entered the intracellular space, as indicated by higher metal accumulation in the central region of the fungi. In contrast, metal signals from CFS and CFS‐G were predominantly localized in peripheral areas, while CFS‐I was similar to the background signal in the control group. ICP‐OES results indicated lower metal content in CFS‐P compared to CFS at the same mass concentration, implying that the higher intracellular deposition of CFS‐P was due to enhanced fungal uptake, rather than different metal content (Figure S6). These findings confirm that the choice of capping agent strongly influences the morphology and cellular uptake of CuFeSe_2_, which are critical for fungal inhibition. Interestingly, the negative zeta potential of CFS‐P does not hinder the interaction with the negatively charged fungal cell wall, suggesting that the nonspecific electrostatic interaction is not the dominant determinant of the activity in this system. Importantly, the unique shape and directional crystal growth of CFS‐P may contribute to its fungicidal efficacy by exposing specific crystal facets, which will be discussed in the following sections.

### 2.3 Cell Wall Mannan as a Key Target of CFSP

**FIGURE 4:**
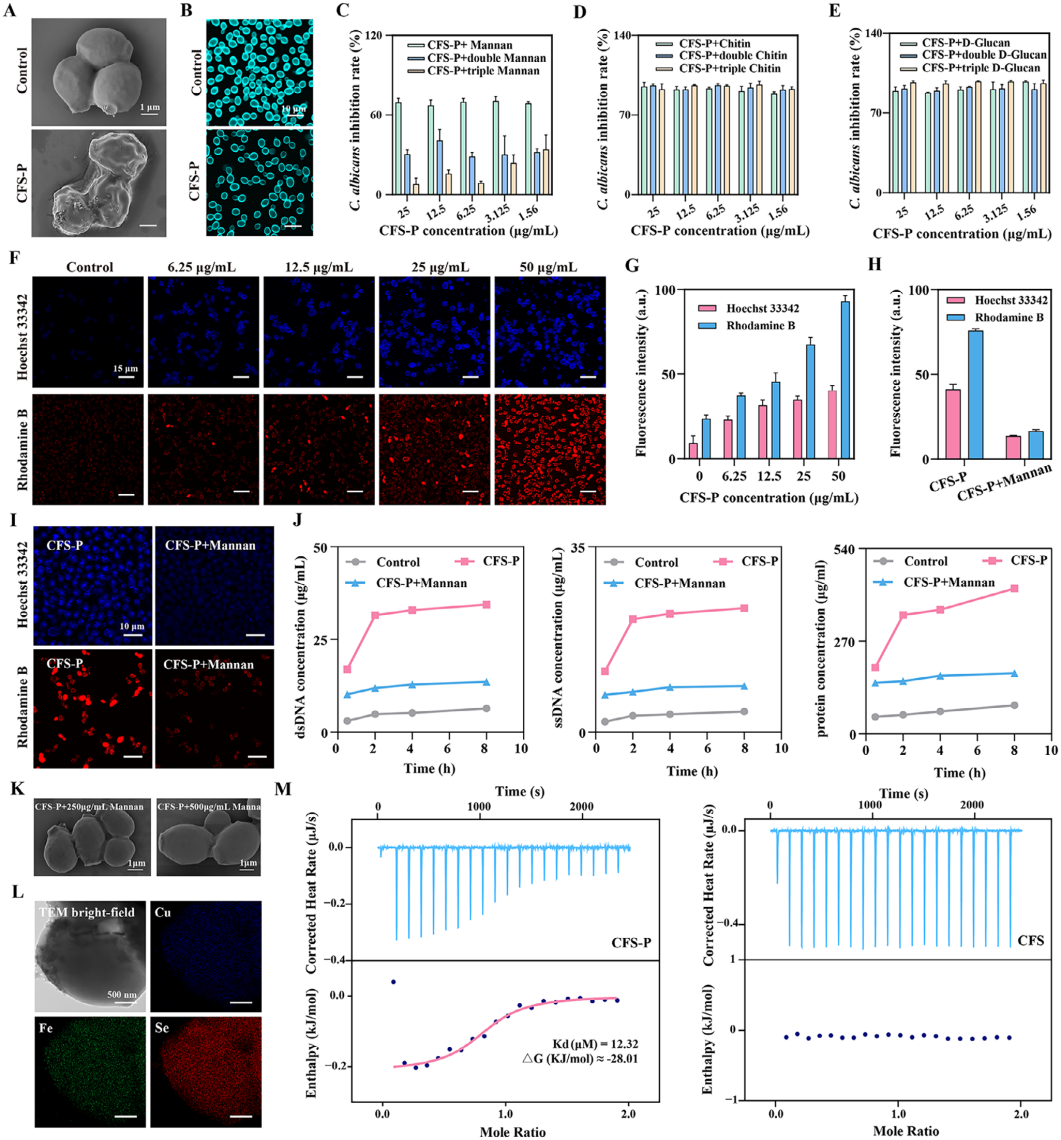
The interaction of CFS‐P with cell wall mannan. (A) SEM images of *C. albicans* treated with PBS or CFS‐P. (B) Fluorescence images of *C. albicans* after co‐incubation with PBS or CFS‐P and stained with Calcofluor White. (C–E) Inhibitory rates of CFS‐P against *C. albicans* after pre‐incubation of CFS‐P with mannan, chitin, and glucan one‐, two‐, and three‐fold of CFS‐P concentrations. (Data were presented as mean ± SD, *n* = 3) (F) Fluorescence images of *C. albicans* incubated with different concentrations of CFS‐P and stained with Hoechst 33342 and Rhodamine B. (G) Semi‐quantitative analysis of Hoechst 33342 and Rhodamine B fluorescence intensity. (Data were presented as mean ± SD, *n* = 3) (H, I) Fluorescence images (I) and semi‐quantitative analysis (H) of Hoechst 33342 fluorescence intensity of *C. albicans* treated with CFS‐P and CFS‐P blocked by mannan. (Data were presented as mean ± SD, *n* = 3) (J) Leakage of protein, single‐stranded DNA, and double‐stranded DNA into the supernatant of cultures treated under different treatments at 0.5, 2, 4, and 8 h. (Data were presented as mean ± SD, *n* = 3) (K) SEM images of *C. albicans* after co‐incubation with CFS‐P that was blocked by excessive mannan. (L) TEM image and EDS mapping of *C. albicans* treated with CFS‐P that was blocked by mannan. (M) Isothermal titration calorimetry (ITC) profiles of the binding of CFS‐P and CFS with mannan.

Based on the above results, we hypothesize that CFS‐P interacts with fungi at the molecular level, involving key fungal cell wall components and specific crystal facets of CFS‐P. To explore the hypothesis, we first investigated whether the cell wall components were related to the fungicidal activity of CFS‐P, given that the cell wall is the primary barrier to drug uptake. CFS‐P induced fungal cell shrinkage (Figure 4A) and reduced cell wall density, as shown by attenuated fluorescence in Calcofluor White staining and semi‐quantitative analysis (Figure 4B and Figure S7), suggesting that CFS‐P may disrupt dense polysaccharide networks in the cell wall. Since mannan constitutes the outermost cell wall structure, we speculate that CFS‐P binds to mannan chains with high molecular avidity. To validate this, we performed blocking experiments by saturating the possible active sites in CFS‐P with exogenous mannan, and the biological effects were compared. In the inhibition test, pre‐incubation with mannan markedly reduced the antifungal activity of CFS‐P when the mannan concentration was 2 times higher than CFS‐P (the double Mannan group in Figure 4C). In contrast, chitin and dextran, the other major cell wall components located near the cell membrane, had little effect on CFS‐P activity (Figure 4D,E). The structurally related mannose‐containing polysaccharides, including glucomannan and galactomannan, were also used to test the blocking effect. Neither glucomannan nor galactomannan reduced the antifungal activity of CFS‐P against *Candida* (Figure S8A,B). In *Candida*, the outward‐projecting fibrillar region is formed mainly by *N*‐mannan outer chains extending from mannoproteins. This outer fibrillar domain is more mannan‐dominant in composition than the more basal, protein‐proximal glycoprotein region [41], and it is structurally more similar to mannan than galactomannan or glucomannan.

We have also preliminarily tested the efficacy of CFS‐P against *Aspergillus terreus*, a mycotic fungal strain featuring galactomannan as the major mannan‐related component in the cell wall. The inhibition effect was relatively weak (Figure S8C), with moderate reduction of the colony number and size at 25 µg/mL, a concentration that could potently inhibit *Candida* species. One possible explanation for this difference is the variation in the composition and abundance of cell wall mannans in Aspergillus compared with *Candida* species. For Aspergillus, the mannan‐related content is a minor composition in the cell wall [42], which is about 4% amount [43]. Candida species have much more abundant mannan contents, accounting for 40% of the cell wall carbohydrate [44].

Therefore, these data support that CFS‐P may preferentially interact with exposed N‐mannan‐rich outer wall structures in *Candida*, rather than the modified mannose, and the abundance of mannan in fungal cell walls may influence the activity.

Then, the blocking effect of exogenous mannan on the interaction of CFS‐P with the cell wall was examined. Under normal treatment conditions, CFS‐P increased cell wall permeability, as shown by enhanced intracellular penetration of Hoechst and Rhodamine dyes with increasing CFS‐P concentration (Figure 4F,G). When mannan‐saturated CFS‐P was used, dye uptake decreased sharply, as indicated by confocal imaging (Figure 4I) and semi‐quantitative analysis (Figure 4H). In addition, CFS‐P treatment led to abnormal efflux of nucleic acids and intracellular proteins during the 8‐h quantification of these components in the culture medium, whereas no such loss was observed when CFS‐P was blocked by mannan (Figure 4J).

Moreover, SEM imaging showed that mannan‐saturated CFS‐P failed to induce cell wall shrinkage and exhibited minimal material adhesion (Figure 4K). Elemental mapping (Figure 4L) also revealed decreased intracellular uptake of CFS‐P after mannan addition, which only exhibited a background signal, in contrast to the central localization of the metals in fungi treated CFS‐P without exogenous mannan (CFS‐P group in Figure 3G). Isothermal titration calorimetry (ITC) indicated a lower Kd value for the CFS‐P–mannan interaction compared with CFS, the pristine CuFeSe_2_ (Figure 4M and Figure S9). Together, these results clearly demonstrate that the association of CFS‐P with mannan directly causes cell wall abnormalities, enhanced nanocrystal internalization, and fungal inhibition.

### 2.4 Specific Facets of CFS‐P Mediate Fungal Binding

**FIGURE 5:**
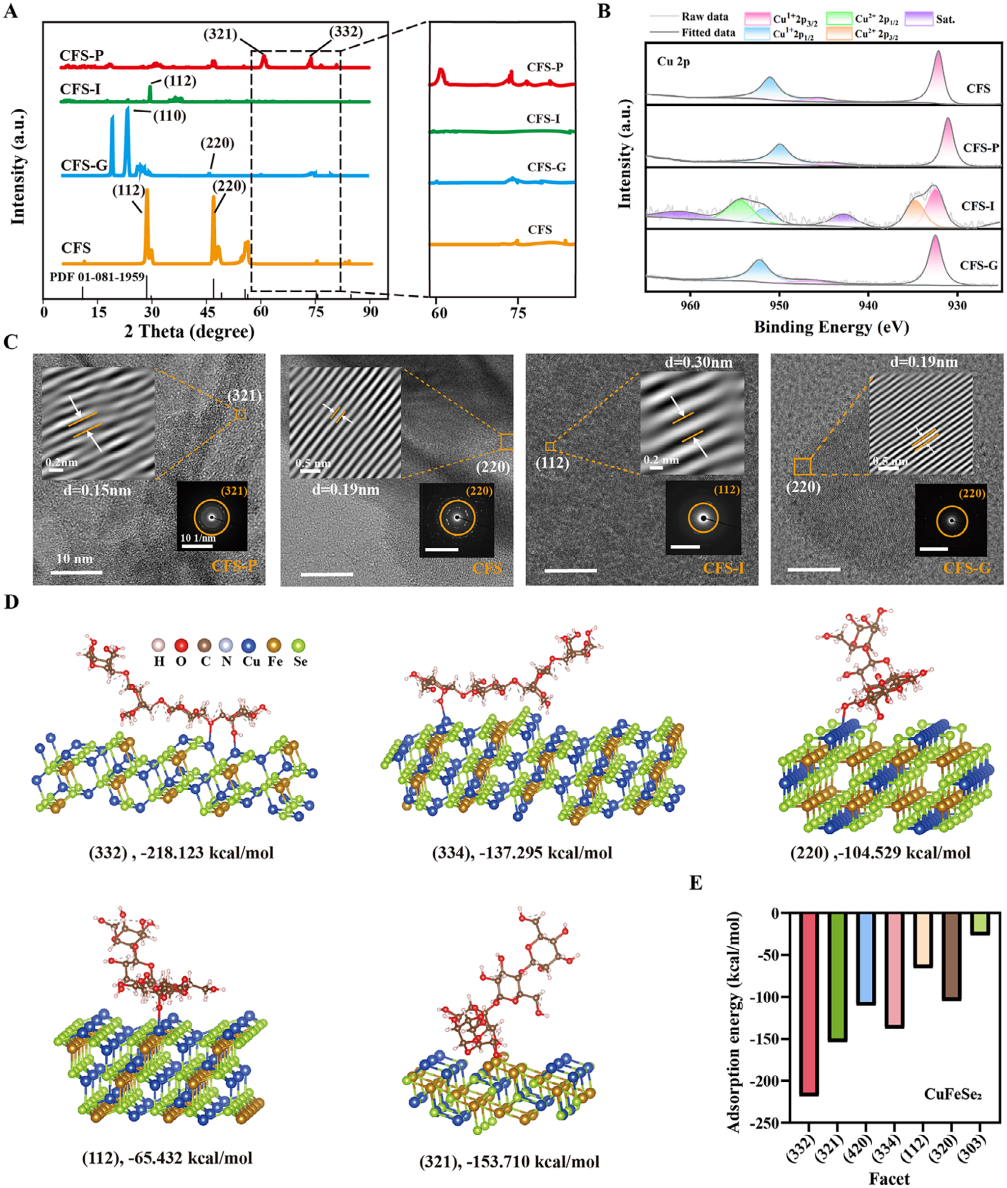
Identification of specific facets of CFS‐P and adsorption energies with mannan. (A) X‐ray diffraction (XRD) patterns of CFS‐P, CFS‐I, CFS‐G, and CFS. (B) X‐ray photoelectron spectroscopy (XPS) spectra of the Cu 2p orbital for CFS‐P, CFS‐I, CFS‐G, and CFS. (C) High‐resolution transmission electron microscopy (HRTEM) images analyzing the crystal structure and interplanar spacing of CFS‐P, CFS‐I, CFS‐G, and CFS nanomaterials. (D) Visualization of specific adsorption sites and their corresponding adsorption energies on different crystal planes of CuFeSe_2_. (E) Calculated adsorption energies for CuFeSe_2_ with different adsorption configurations.

The identification of mannan as a key target of CFS‐P prompted us to investigate the structural features of CFS‐P responsible for mannan avidity. In wet‐chemical synthesis, capping agents significantly influence crystal growth by altering surface energy and facet exposure [45]. X‐Ray Diffraction (XRD) patterns revealed the crystalline phases of the four differently capped crystals (Figure 5A), and X‐ray photoelectron spectroscopy (XPS) validated the composition and valence states of each element in these nanocrystals (Figure 5B and Figure S10). Compared to the pristine CuFeSe_2_ (CFS), the other three crystals showed weaker diffraction intensities, primarily due to polymer masking. All diffraction peaks matched the reference pattern for tetragonal eskebornite CuFeSe_2_ (PDF 01‐081‐1959). Notably, for CFS‐P, the major difference was the occurrence of additional peaks corresponding to high‐index facets, including (321), (332), (334), and (420), with (321) and (332) planes showing obvious diffraction signals. The texture coefficient (TC), calculated based on the quantitative analysis of the XRD data, confirmed the specific regulatory effect of PVP on the crystallization pathway of the material. As shown in Figure S11, among all tested samples, only CFS‐P exhibited a pronounced preferential orientation of the (321) plane, with the TC (321) reaching as high as 3.85, while the (332) facet was exposed but did not show preferential orientation. In contrast, the pristine crystal (CFS) and the other modified control groups (CFS‐I and CFS‐G) all showed negligible TC values for the (321) and (332) planes. HRTEM revealed a lattice spacing of 0.15 nm in CFS‐P, corresponding to the (321) plane (Figure 5C). Selected area electron diffraction (SAED) rings further confirmed interplanar distances matching the (321) planes. HRTEM and SAED data for CFS, CFS‐G, and CFS‐I aligned well with their respective XRD peaks.

**FIGURE 6:**
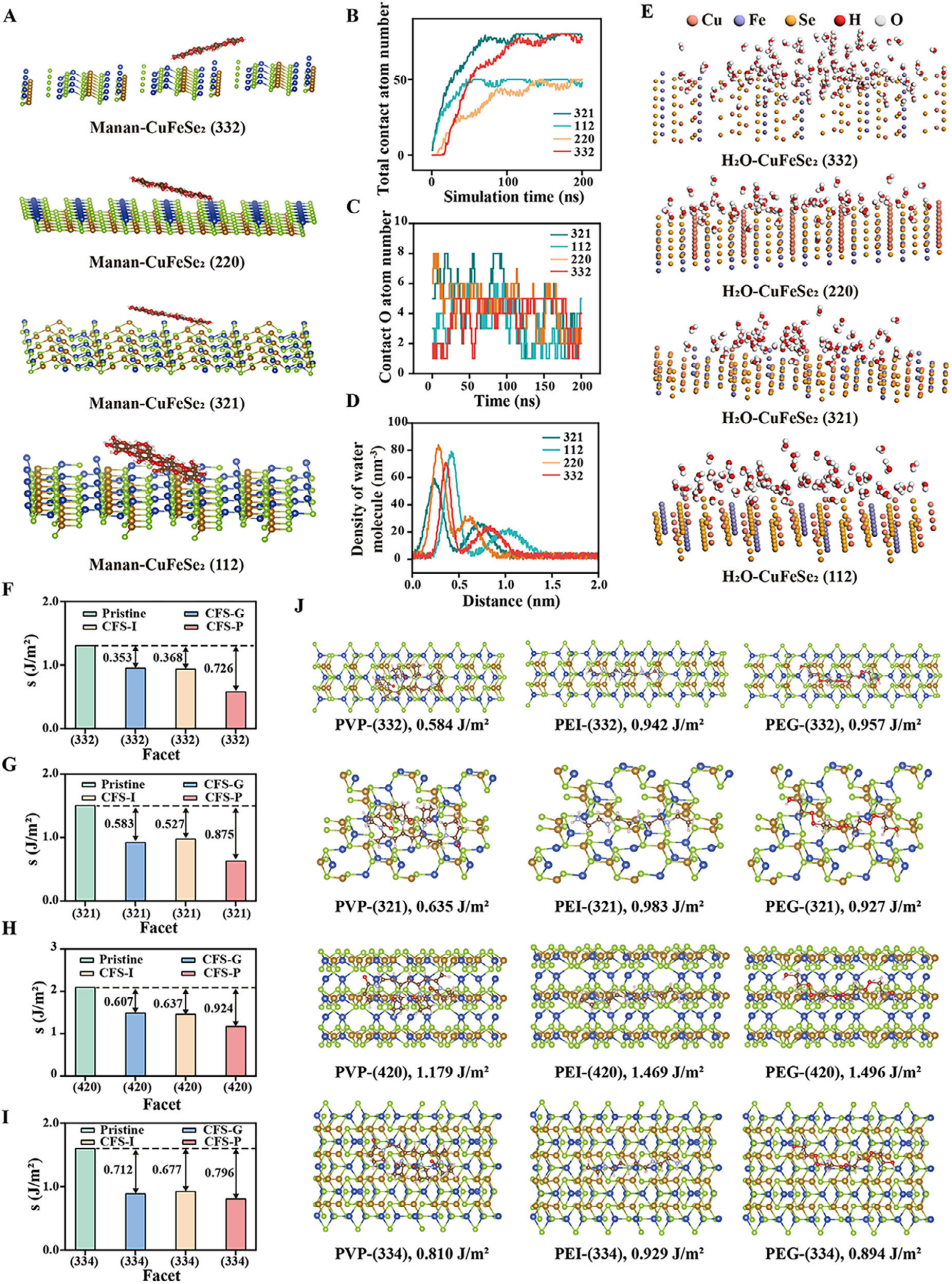
Molecular dynamics simulations and comparison of surface energy reduction. (A) Molecular dynamics simulation snapshots of different crystal planes of CuFeSe_2_ and mannan. (B) Number of mannan atoms bound to different crystal planes of CuFeSe_2_. (C) Number of oxygen atoms in contact with different crystal planes of CuFeSe_2_. (D) Quantified density of water molecules adsorbed on different crystal planes of CuFeSe_2_. (E) Molecular dynamics simulation configurations of water molecules (H_2_O) adsorbed on different crystal planes of CuFeSe_2_. (F–I) Changes in surface energy for different crystal planes of CuFeSe_2_ modified with different capping agents. (J) Configurations and surface energies for different capping agents on different crystal planes.

To determine whether the high‐index facets unique to CFS‐P are involved in fungal interactions, we performed density functional theory (DFT) simulations to study the binding of different crystal facets to mannan. In addition to CFS‐P‐specific facets, typical facets of CuFeSe_2_, including (112), (220), and (303), were also included for comparison. The high‐index facets of CFS‐P, (321), (332), (334), and (420), showed significantly lower interaction energies with mannan compared to the facets of other crystals, indicating more favorable adsorption (Figure 5D,E, and Figure S12A,B). These results suggest that the association between CFS‐P and mannan may depend on the specifically exposed crystal facets. To further specify which facet is the most favorable for mannan binding, molecular dynamics (MD) simulations were conducted. The representative facets, including 2 high‐index facets, (332) and (321), and 2 common facets, (220) and (112), were used to establish the structures for the simulation (Figure 6A). The number of total atoms of mannan in direct contact with the 4 facets was illustrated in Figure 6B. The mannan atoms in contact with (321) facet increased most during the simulation at 50 ns, compared to (332), (220), and (112) facets. At 200 ns, the contacted atom number became similar between (321) and (332) planes, but still evidently larger than that of (220) and (112) planes. The number of oxygen atoms in contact with different facets, which were largely originated from mannan, was higher for (321) plane in the first 100 ns, compared with other facets (Figure 6C), suggesting more stable absorption. Moreover, MD simulation showed that the density of water molecules was the lowest for (321) facet in the first solvation shell (Figure 6D,E). The generally loose water arrangement in (321) plane may facilitate easier replacement of mannan to the water molecules [33].

In addition, MD simulations of the interaction of the characteristic (321) and (332) facets with β‐glucan, chitin, glucomannan, and galactomannan were performed. The results showed that, relative to mannan, all these polysaccharides exhibited substantially weaker interactions with these facets (Figure S13). Together, these results support that the facet‐dependent recognition of CFS‐P is strongest for mannan rather than being a general feature for all cell‐wall polysaccharides.

In general, colloidal nanocrystal growth follows a kinetic process in which high‐energy, high‐index facets vanish quickly, leaving slower‐growing low‐index facets to dominate the crystal surface [46]. To explain why high‐index facets are preferentially exposed in PVP‐capped CuFeSe_2_ rather than in PEG‐ or PEI‐capped variants, we used DFT to assess changes in surface energy upon association with each capping agent. All three capping agents reduced the surface energies of the four facets to some extent, but the most substantial reductions were observed with PVP (Figure 6F–J, and Figure S12C–G). Thus, the exposure of high‐index facets in CFS‐P is likely due to sufficient surface energy reduction by PVP. These results, consistent with XRD analysis, provide a theoretical basis for facet engineering of CuFeSe_2_ nanocrystals to form mannan‐adhesive high‐index planes. Furthermore, the data highlight the potential for future research to precisely control directional crystal growth under the ambient aqueous condition, and tailor functional facets for enhanced biomedical efficacy.

### 2.5 Vacuolar Destruction by CFS‐P

**FIGURE 7:**
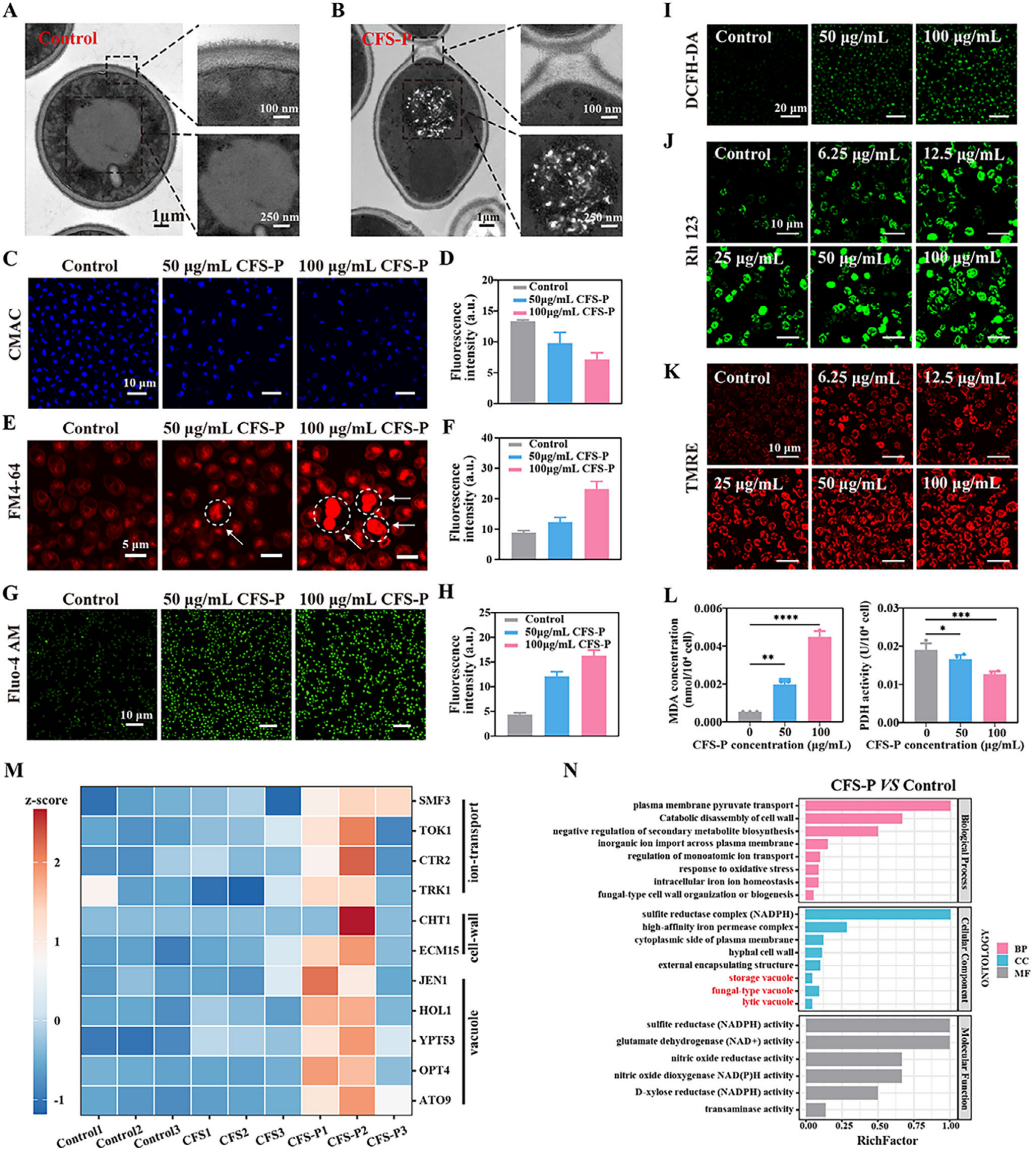
Vacuolar destruction by CFS‐P. (A, B) SEM images of *C. albicans* treated with PBS control and CFS‐P. (C, D) Fluorescence images (C) and quantitative analysis (D) of *C. albicans* incubated with different concentrations of CFS‐P and stained with CMAC. (Data were presented as mean ± SD, *n* = 3) (E, F) Fluorescence images (E) and quantitative analysis (F) of *C. albicans* incubated with different concentrations of CFS‐P and stained with FM4‐64. (Data were presented as mean ± SD, *n* = 3) (G, H) Fluorescence images (G) and quantitative analysis (H) of *C. albicans* incubated with different concentrations of CFS‐P and stained with Fluo‐4 AM. (Data were presented as mean ± SD, *n* = 3) (I) Fluorescence images of *C. albicans* incubated with different concentrations of CFS‐P and stained with DCFH‐DA. (J) Fluorescence images of *C. albicans* incubated with PBS or CFS‐P and stained with Rhodamine 123. (K) Fluorescence images of *C. albicans* incubated with PBS or CFS‐P and stained with TMRE. (L) Measurement of malondialdehyde (MDA) content and pyruvate dehydrogenase (PDH) activity in *C. albicans* incubated with different concentrations of CFS‐P. (Data were presented as mean ± SD, *n* = 3, ****p < 0.0001, ***p < 0.001, **p < 0.01, and *p < 0.05) (M) Heatmap of key DEGs changes by CFS‐P treatment compared with CFS and Control. (N) GO annotation analysis of DEGs in *C. albicans* (CFS‐P VS control).

Having established that CFS‐P associates with fungi primarily through crystal–mannan interaction, we next explored the intracellular fate of CFS‐P and subsequent biological changes. Interestingly, TEM of fungal sections revealed multiple cavities in the vacuoles of CFS‐P‐treated fungi, along with loosened cell walls (Figure 7A,B). Given the previous EDS results showing CFS‐P accumulation in the central vacuolar region (Figure 3G), we speculate that CFS‐P enters and disrupts fungal vacuoles following mannan‐mediated uptake. We then performed functional staining experiments using vacuole‐specific dyes CMAC (7‐amino‐4‐chloromethylcoumarin) and FM4‐64 to assess vacuolar damage. CMAC forms a conjugate via glutathione S‐transferase and is transported into the vacuole by glutathione pumps on the vacuolar membrane, and its accumulation reflects vacuolar membrane integrity. CFS‐P treatment significantly reduced CMAC fluorescence, indicating possible vacuolar membrane damage (Figure 7C,D). FM4‐64, a lipophilic dye that labels endocytic and exocytic membranes [47], showed evenly distributed fluorescence on membrane‐like organelles in control fungi (Figure 7E). In CFS‐P‐treated cells, however, largely diffused red signals without clear boundaries were observed, suggesting membrane collapse (Figure 7E,F). These observations became more evident with higher CFS‐P concentration.

Vacuoles serve as the primary calcium storage sites in fungi, and their disruption may induce cellular calcium overload. CFS‐P treatment strongly enhanced fluorescence of the calcium indicator Fluo‐4 AM, confirming elevated intracellular Ca^2^^+^ (Figure 7G,H). The intracellular ROS level was also elevated after CFS‐P treatment, possibly resulting from excess Ca^2^^+^. Since calcium phosphate Ca_3_(PO_4_)_2_ is the main form of calcium storage in fungal vacuoles, we used it as a model to test whether CFS‐P induces Ca^2^^+^ release. ICP‐OES detected gradual Ca^2^^+^ release from Ca_3_(PO_4_)_2_ with increasing CFS‐P concentration, suggesting that CFS‐P may not only disrupts vacuolar structure but also chemically mediates Ca^2^^+^ release (Figure S14). To determine whether CFS‐P generates reactive oxygen species (ROS) upon interaction with *C. albicans*, we stained the yeast with the fluorescent probe DCFH‐DA. While no obvious fluorescence was detected in the untreated control, signifying baseline ROS levels, the CFS‐P‐treated fungal cells exhibited a significant increase in green fluorescence, indicating intracellular ROS production (Figure 7I and Figure S15), which was likely to be a downstream consequence of cellular or organelle injury induced by CFS‐P.

Mitochondria are known to respond to elevated calcium levels, which can trigger cell death and energy production dysfunction [48]. Mitochondrial staining was performed to assess whether CFS‐P induces mitochondrial impairment. Very interestingly, treatment with CFS‐P resulted in a dose‐dependent fluorescence increase in both Rhodamine 123 (Figure 7J and Figure S16A) and tetramethylrhodamine ethyl ester (TMRE) (Figure 7K and Figure S16B). The fluorescence intensity in CFS‐P‐treated cells also showed a positive correlation with increasing CFS‐P concentration. These results suggest that CFS‐P treatment led to abnormally increased mitochondrial membrane potential (ΔΨm) in *C. albicans*, different from mostly reported decreased membrane potential in damaged mitochondrion of mammalian cells [49, 50]. Significant decrease of ATP production was observed after CFS‐P treatment, further supporting the mitochondrial dysfunction induced by CFS‐P (Figure S17). To further examine the temporal relationship between vacuolar damage and mitochondrial dysfunction, the time‐course staining experiments were performed to monitor mitochondrial polarization and vacuolar damage at 0.5, 1, 2, and 4 h after CFS‐P treatment. Interestingly, abnormal mitochondrial hyperpolarization was already evident at 0.5 h, whereas clear vacuolar functional impairment became apparent after 1 h (Figure S18). These findings preliminarily suggest that detectable vacuolar dysfunction may occur after mitochondrial injury rather than preceding it.

Mitochondrial damage was accompanied by significantly reduced pyruvate dehydrogenase (PDH) activity and increased lipid peroxide (LPO) accumulation as indicated by elevated MDA concentration (Figure 7L). These changes not only confirm mitochondrial dysfunction but also suggest the occurrence of cuproptosis, a copper‐dependent cell death pathway [51].

Transcriptomic sequencing was performed to further validate and explore the biological mechanisms of CFS‐P. The number of differentially expressed genes (DEGs) is summarized in Figure 7M and Figure S19. Consistent with our observations, vacuole‐related genes were significantly upregulated in the CFS‐P group compared to the control and CFS groups. Genes associated with cell wall and ion transport were also upregulated. Gene Ontology (GO) annotation analysis revealed functional gene changes in biological process (BP), cellular component (CC), and molecular function (MF) categories (Figure 7N and Figure S19). Both CFS‐P and CFS induced genetic changes related to metal transport, cell wall, and respiratory chain, possibly due to material adhesion and limited metal internalization. Notably, only CFS‐P caused significant alterations in vacuole‐related genes. GO results align with our observation that CFS‐P acts on vacuoles after fungal uptake. CFS‐P also induced more pronounced changes in metal homeostasis genes, likely due to its enhanced fungal uptake compared to CFS. Taken together, CFS‐P resulted in more alterations toward metal homeostasis, which could be attributed to the enhanced fungal uptake of CSF‐P compared with CSF. The results suggest that the vacuole damage may account for the cell death after the interaction of CFS‐P and the fungi.

### 2.6 In Vivo Antifungal Efficacy

**FIGURE 8:**
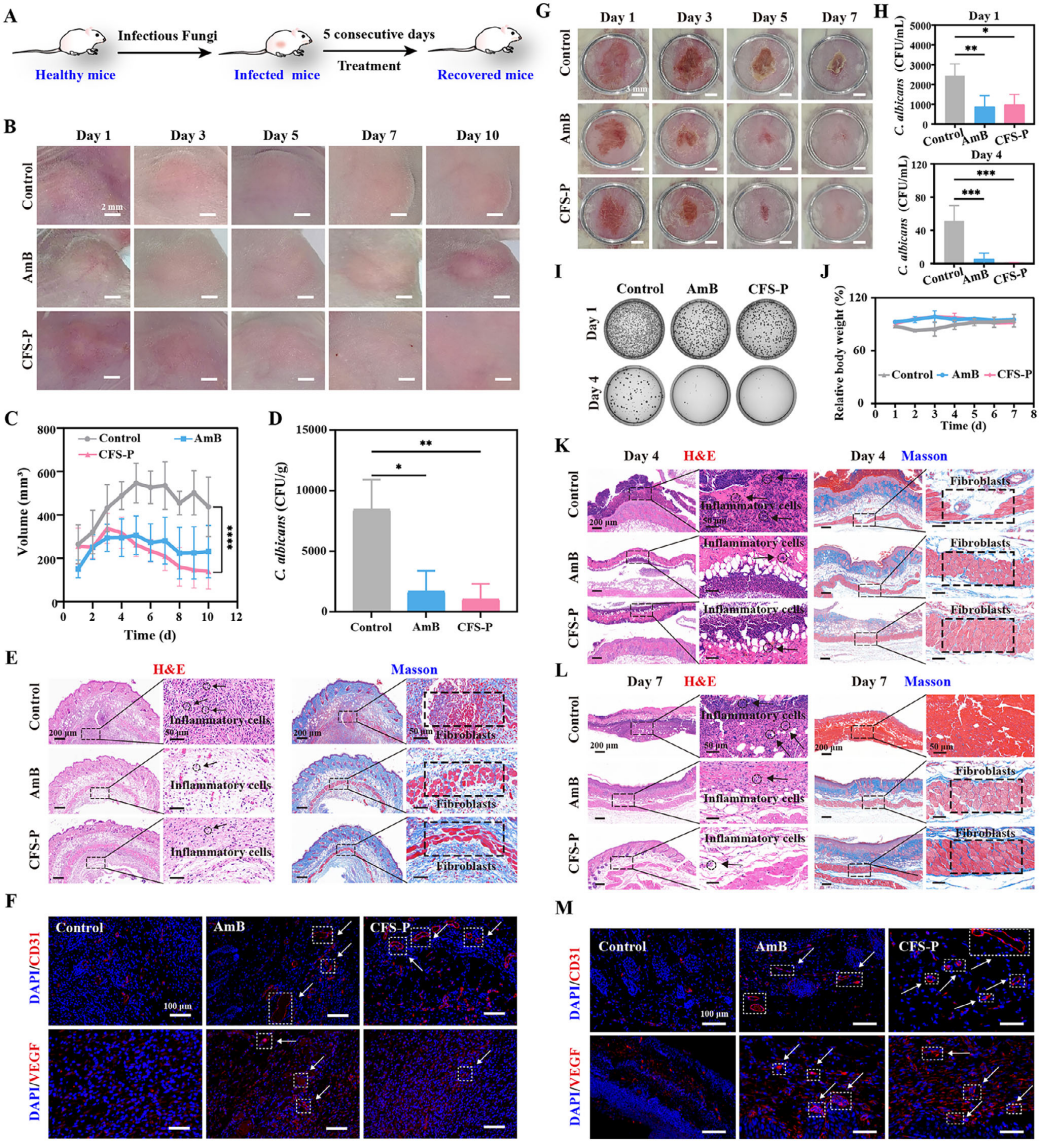
In vivo antifungal efficacy. (A) Schematic diagram of the deep‐skin fungal infection treatment. (B) Photographs of mouse *Candidiasis* foci on days 1, 3, 5, 7, and 10 after different treatments. (Data were presented as mean ± SD, *n* = 6) (C) Lump size curves of mice before and after treatment in the deep‐skin infection model. (Data were presented as mean ± SD, *n* = 6. ****p < 0.0001) (D) Colony counts from wound tissues of mice on day 10 after different treatments (Data were presented as mean ± SD, *n* = 5, **p < 0.01, and *p < 0.05) (E) H&E and Masson staining of the infected skin tissues on day 10. (F) CD31 and VEGF immunofluorescence staining of the infected skin tissues on day 10. (G) Photographs of mouse skin wounds on days 1, 3, 5, and 7 after different treatments. (Data were presented as mean ± SD, *n* = 6) (H) Photographs of plates from colony counting of from mouse wound tissues on days 1 and 4 after different treatments. (Data were presented as mean ± SD, *n* = 4) (I) Colony counts of wound tissues on days 1 and 4 after different treatments (Data were presented as mean ± SD, *n* = 4. ***p < 0.001, **p < 0.01, and *p < 0.05). (J) Body weight change curves of mice before and after treatment in the abrasion infection model. (Data were presented as mean ± SD, *n* = 3) (K) H&E and Masson staining of impaired dorsal skin tissues on day 4 after different treatments. (L) H&E and Masson staining of impaired dorsal skin tissues on day 7 after different treatments. (M) CD31 and VEGF immunofluorescence staining of skin tissues from impaired dorsal skins.

The therapeutic efficacy of CFS‐P was evaluated in vivo using deep‐skin mycosis and impaired dorsal skin infection models. The experimental timeline and treatment procedure of the deep‐skin model are illustrated in Figure 8A. The *Candidiasis* foci were imaged over a 5‐day treatment period and monitored again on days 7 and 10 post‐therapy. Compared to control and AmB groups, CFS‐P most effectively alleviated abscesses and reduced edema volume (Figure 8B). Edema in the CFS‐P group nearly resolved by day 10, five days after the final administration. Visual observations were consistent with volumetric measurements, confirming a significant reduction in abscess size (Figure 8C). Quantification of fungal load in skin tissues also showed a sharp decrease after CFS‐P therapy (Figure 8D). H&E and Masson staining of tissue samples from each group were used to assess pathological conditions. CFS‐P markedly reduced inflammatory cell infiltration (red arrows in enlarged areas) and increased collagen fiber deposition (Figure 8E). The overall therapeutic efficacy of CFS‐P was comparable to that of AmB. Importantly, CFS‐P significantly promoted the formation of vascular structures, as indicated by CD31 staining. Notably, the vascular recovery effect of CFS‐P surpassed that of AmB. Expression of VEGF, a key angiogenesis‐stimulating factor, was also visualized in tissue samples, with the CFS‐P group showing the most abundant VEGF protein (Figure 8F). The enhanced CD31 and VEGF expression may be attributed to the well‐established role of copper in promoting angiogenesis [52].

In mice with cutaneous fungal infection on damaged dorsal skin, CFS‐P also demonstrated superior therapeutic efficacy, as evidenced by accelerated skin repair within 7 days (Figure 8G). Plate cultures of skin tissue homogenates showed nearly complete fungal eradication by day 4 with CFS‐P, comparable to the AmB positive control (Figure 8I). Colony quantification confirmed these observations (Figure 8H). Pathological examination of treated skin revealed that CFS‐P reduced inflammatory burden at day 4 post‐treatment and achieved nearly complete skin recovery by day 7 (Figure 8K,L). In contrast, control groups still exhibited extensive inflammatory cell infiltration and tissue necrosis. Collagen fibers also gradually recovered from day 4 to day 7 in the CFS‐P group, with effects similar to those of AmB. Immunofluorescence imaging validated increased CD31 and VEGF expression in CFS‐P‐treated tissues, indicating vascular normalization (Figure 8M). Moreover, mouse body weights remained stable throughout the treatment (Figure 8J).

### 2.7 Biocompatibility of CFS‐P

**FIGURE 9:**
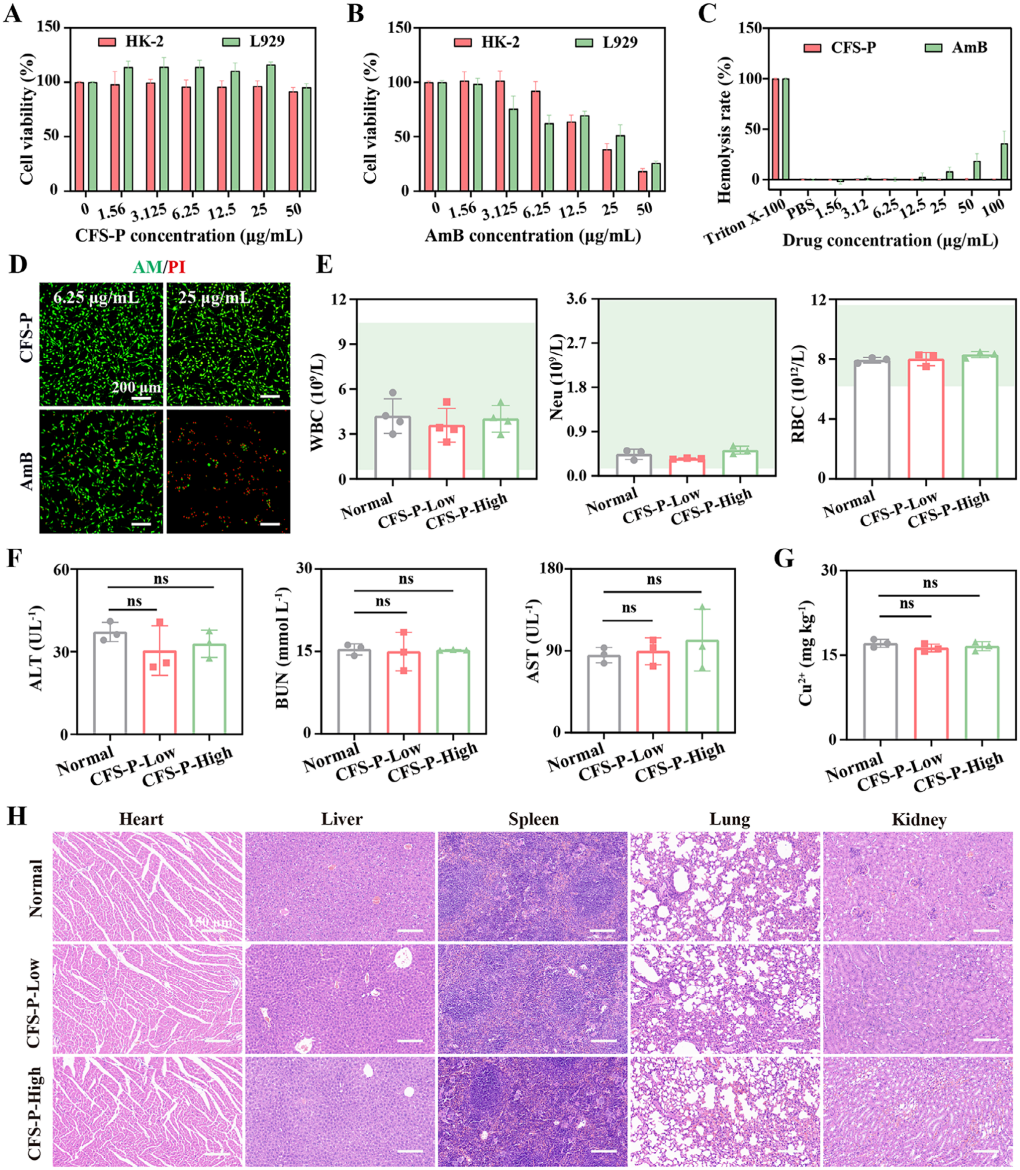
Biocompatibility of CFS‐P. (A) Viability of L929 and HK‐2 cells after treatment with different concentrations of CFS‐P (A) and AmB (B). (Data were presented as mean ± sd, *n* = 5) (C) Hemolysis rate of mouse red blood cells treated with different concentrations of CFS‐P or AmB (Data were presented as mean ± sd, *n* = 3). (D) Calcein‐AM/PI live/dead cell staining fluorescence images of NIH‐3T3 cells after treatment with different concentrations of CFS‐P or AmB. (E) Complete blood count analysis of ICR mice on day 3 after intravenous injection of CFS‐P. (Data were presented as mean ± sd, *n* = 3) (F) Serum biochemical analysis of ICR mice on day 3 after intravenous injection of CFS‐P. (Data were presented as mean ± SD, *n* = 3) (G) Copper (Cu) content in the livers of ICR mice on day 3 after intravenous injection of CFS‐P. (Data were presented as mean ± SD, *n* = 3) (H) H&E staining of heart, liver, spleen, lung, and kidney tissues from ICR mice on day 3 after intravenous injection of CFS‐P.

A prerequisite for the in vivo application of CFS‐P is the safety in mammalian cells. As noted, CFS‐P targets the cell wall and vacuole, the organelles unique to fungi, suggesting fungal specificity and good mammalian cell tolerance. AmB, the first‐line antifungal, inhibits fungal growth by targeting ergosterol in the cell membrane but also causes severe toxicity to normal cells. We compared the toxicity of CFS‐P and AmB in HK‐2 and L929 cells. CFS‐P showed excellent biocompatibility at concentrations ranging from 1.56 to 50 µg/mL (Figure 9A). In contrast, AmB drastically reduced cell viability at 12.5 µg/mL, and most cells died at 50 µg/mL (Figure 9B). Live/dead staining provided a visual comparison (Figure 9D). At 6.25 µg/mL, both AmB and CFS‐P groups showed predominantly green AM fluorescence and minimal PI signal, indicating low toxicity. At 25 µg/mL, cells in the CFS‐P group remained largely viable, whereas the AmB group showed extensive PI staining, indicating widespread cell death. At higher concentrations, AmB caused significant hemolysis, whereas CFS‐P was compatible with red blood cells even at 100 µg/mL (Figure 9C). CFS‐P also showed favorable compatibility with macrophages, the important host immune cells (Figure S20). We further evaluated the biosafety of CFS‐P after intravenous injection. Importantly, key hematological and biochemical indices showed no significant changes compared to healthy mice (Figure 9E,F and Figure S21). Notably, the CFS‐P‐high group received 10 mg/kg CFS‐P, a particularly high dose for tail vein injection. With such a high amount of CFS‐P administration, no excessive metal accumulation in the liver was found compared to healthy controls (Figure 9G). Additionally, H&E staining revealed no obvious acute pathological changes (Figure 9H). Our current in vivo data have provided the initial evidence of favorable short‐term tolerance, including stable hematological and biochemical indices, absence of obvious acute histopathological abnormalities, and no excessive liver copper accumulation after intravenous injection. However, the long‐term toxicity over 90 days, and the pharmacokinetic characteristics of CFS‐P need further exploration to fully evaluate the translational potential, especially for the treatment of invasive fungal infection. These specific investigations will be carried out in our future study that focuses on the further application of CFS‐P.

## 3 Conclusion

In this study, we elucidated facet‐dependent, polysaccharide‐targeting fungicidal mechanism of a semiconductor nanocrystal, CFS‐P, a PVP‐capped CuFeSe_2_ nanocrystal with exceptional fungicidal activity and biocompatibility. Our findings reveal a novel “wall–vacuole” dual‐organelle targeting strategy, which distinguishes CFS‐P from conventional antifungal agents, including nano‐materials. Distinct from the mostly reported “nanozyme” catalytic activity that inactivates microbial through the production of free radicals, this study discovered a novel “fungal‐facet” interface focusing on the crystal interaction with cell wall mannan.

While facet engineering has been widely applied in catalysis and energy conversion, its role in mediating fungal inhibition has not been reported yet. Our integrated experimental and computational analyses demonstrate that the specific high‐index facets exposed in CFS‐P exhibit strong affinity for mannan, the major polysaccharide in the fungal outer cell wall. Notably, PVP reduces the surface energy of high‐index facets more effectively, facilitating their exposure conducive to mannan binding. This interaction not only increases cell wall permeability but also promotes efficient nanocrystal internalization into the vacuolar compartment, leading to organelle dysfunction, homeostasis disruption, and fungal death. Although our current data do not demonstrate exclusive recognition of a single defined mannan epitope, the results support the conclusion that mannan‐containing outer‐wall components are major contributors to CFS‐P binding and uptake, which is supported by the mannan blocking experiments, the ITC results, and the facet‐dependent computational analyses. Recent biophysical and structural studies of fungal cell walls have revealed the mannan fibril structure of the outer cell wall and provided a relatively precise three‐dimensional model demonstrating the mannan distribution in the fibrillar architecture. For *Candida* species, most of the mannans from the mannoproteins radiate out to form the fibrils rather than being embedded within basal layers of the cell wall [14, 41]. Therefore, in our study, CFS‐P most probably interacts with the radiated mannose in the first place. The solid‐state NMR studies have also emphasized that fungal cell walls contain structurally polymorphic and dynamically heterogeneous carbohydrate domains in their native state, including mannan‐ or galactomannan‐containing assemblies in *Candida* and *Aspergillus* [53, 54]. In this study, mannan is treated as a relatively simple polysaccharide target, which is generally rational when dealing with the outermost fibrillar mannan in *Candida*. More efforts in the future should be invested in the more specific recognition of polysaccharides motifs by the semiconductor nanocrystals. Further, in future work, we will correlate the fungicidal activities of CFS‐P with the cell‐wall polysaccharide composition and surface organization of multiple fungal species to further extend the antifungal spectrum.

The other unique feature of CFS‐P is the distinct intracellular activity. Upon internalization, CFS‐P accumulates in the vacuole, inducing multiple cavities. The vacuole damage has rarely been reported in other super‐molecular structures. The subsequent calcium dyshomeostasis and mitochondrial damage are much likely to be associated with vacuolar disruption. Interestingly, the cuproptosis‐like cell death pathway may also occur, which is validated by lipid peroxidation and suppression of pyruvate dehydrogenase. It should be pointed out that the observed intracellular Ca^2^^+^ increase is unlikely to be explained solely by direct chemical interaction with calcium phosphate. Another plausible interpretation is that the vacuolar dysfunction induced by CFS‐P contributes substantially to Ca^2^^+^ dyshomeostasis. In fungi, the vacuole is the major intracellular Ca^2^^+^ storage organelle, and vacuolar Ca^2^^+^ homeostasis depends on both vacuolar acidification and vacuolar Ca^2^^+^ transport systems on the vacuolar membrane [55]. Therefore, once CFS‐P disrupts vacuolar structure and function, the ability of the vacuole to sequester and buffer Ca^2^^+^ may be impaired, leading to elevated cytosolic Ca^2^^+^. In addition, mitochondrial dysfunction may further aggravate this process by reducing ATP availability and thereby compromising ATP‐dependent vacuolar functions, including maintenance of the proton gradient. More importantly, unlike polyene drugs that target ergosterol with off‐target toxicity, CFS‐P exploits structural and organellar differences between fungi and mammalian cells, enabling selective fungal eradication and promising clinical translation potential.

In conclusion, this work not only deciphers the facet‐driven antifungal mechanism of CFS‐P but also establishes a new paradigm for the rational design of biocompatible semiconductor antibiotics to overcome drug resistance and host toxicity. Future efforts will focus on deepening the understanding of the facet‐microbial interface and exploring its applicability against a broader spectrum of pathogens. Specifically, facet engineering can be integrated with pathogen‐associated molecular recognition beyond the CuFeSe_2_ system. Future studies may fully leverage theoretical calculations, artificial intelligence, and related techniques to identify “active facets” that target microorganism‐specific structures, thereby enabling the screening of nanocrystals with high affinity for multi‐microorganisms. For example, lipopolysaccharide (LPS), as the characteristic component of the Gram‐negative bacterial cell wall, is an ideal target for facet‐encoded recognition. Once internalized by microbial cells, the nanocrystals may disrupt microbial homeostasis through their diverse physiochemical properties and thereby exert intrinsic antimicrobial activity. The intracellular mechanisms of action also require deeper and more detailed investigation, such as the temporal sequence of vacuolar and mitochondrial damage and their dependency relationship. In addition, materials with active facets may also serve as efficient drug carriers, reducing toxicity while enhancing the efficacy of existing small‐molecule drugs. Future challenges remain in achieving precise control over the crystal facets of water‐soluble nanocrystals and in validating the reliability of virtual facet screening, which will require close interdisciplinary efforts integrating materials science, microbiology, theoretical computation, and artificial intelligence.

## 4 Experimental Section

### 4.1 Materials

Selenium powder, anhydrous copper (II) chloride, anhydrous iron (II) chloride, and polyvinylpyrrolidone (PVP, average molecular weight 8000) were purchased from Shanghai Aladdin Biochemical Technology Co., Ltd. Amphotericin B was purchased from Rhawn. Malondialdehyde (MDA) Content Assay Kit, Pyruvate Dehydrogenase (PDH) Activity Assay Kit, and Hoechst 33342 were purchased from Beijing Solarbio Science & Technology Co., Ltd. Mannan was purchased from Shanghai Acmec Biochemical Technology Co., Ltd. Calcofluor White Stain was purchased from Sigma. *C. albicans* (ATCC10231), *Candida tropicalis (Castellani) Berkhout* (BNCC334135), *Candida glabrata (Anderson) Meyer et Yarrow* (BNCC245220), *Staphylococcus aureus subsp. aureus* (ATCC29213) and *Escherichia coli* (ATCC 25922), and *Aspergillus terreus* (BNCC185690) were purchased from BeNa Culture Collection. Female BALB/c mice (18–25 g) and female ICR mice were acquired from Hainan Fenwei Biotechnology Co., Ltd. All the animal experiments were conducted in compliance with the Animal Management Rules of the Ministry of Health of the People's Republic of China, and approved by the Institutional Animal Care and Use Committee of Hainan University (HNUAUCC‐2021‐00021).

### 4.2 Synthesis of Nanocrystals

#### 4.2.1 Synthesis of Nanocrystal CFS‐P

Selenium powder (21.8 mg) was added to a dry three‐necked flask. Then, 48 mL of ddH_2_O was added, and a stir bar was placed inside. The setup was placed on a magnetic stirrer, and nitrogen gas was purged through the system for 30 min to remove air. Subsequently, sodium borohydride (35 mg) was dissolved in 1 mL of ddH_2_O and injected slowly along the wall of the flask using a 2.5 mL syringe. The reaction proceeded for 2 h until the solution turned clear and colorless. Next, anhydrous copper chloride (18.19 mg), FeCl_2_ (16.21 mg), and PVP (100 mg) were dissolved in 2 mL of ddH_2_O, ultrasonicated for approximately 3 min until fully mixed, and rapidly injected into the reaction system using a 2.5 mL syringe. The reaction continued for 1 h before termination. A small aliquot of the liquid was diluted and observed for clarity; a clear solution indicated successful nanoparticle synthesis. The resulting sample was transferred to a 100 kDa dialysis bag and dialyzed against pure water for 48 h, with one water change. Finally, the sample was transferred to a 50 mL centrifuge tube, lyophilized, and stored in a desiccator.

### 4.3 In Vitro Fungicidal Evaluation of CFS‐P

#### 4.3.1 Minimum Inhibitory Concentration (MIC) Assay

A 96‐well microtiter plate was divided into 4 groups: negative control, treatment, reference, and blank. The negative control group consisted of six replicate wells, each containing 50 µL of liquid YM medium and 50 µL of diluted fungal suspension. The blank group contained six replicate wells, each filled with 100 µL of liquid YM medium. The treatment was designed with 7 concentration gradients, each having 3 replicate wells. Each well in this group first received 50 µL of liquid YM medium, followed by the addition of 50 µL of CFS‐P solution diluted in YM medium (or other drugs) to the three replicate wells of the highest concentration (25 µg/mL). A two‐fold serial dilution was then performed sequentially to decrease the concentration across the wells, resulting in concentrations of 12.5, 6.25, 3.125, 1.56, 0.78, and 0.39 µg/mL. Finally, 50 µL of diluted fungal suspension was added to each well. The reference group also included seven concentration gradients, each with two replicate wells. Each well in this group was initially filled with 50 µL of liquid YM medium, followed by the addition of 50 µL of CFS‐P solution diluted in liquid medium to the highest concentration wells (25 µg/mL). A two‐fold serial dilution was similarly carried out, resulting in concentrations of 12.5, 6.25, 3.125, 1.56, 0.78, and 0.39 µg/mL, after which 50 µL of liquid medium was added to each well. After all treatments, the 96‐well plate was sealed and incubated statically at 30°C for 12 h. The absorbance (OD600) of each well was then measured at a wavelength of 600 nm using a microplate reader, and the data were recorded. Using the average absorbance values of the negative control and blank groups as baselines, the average absorbance for each concentration gradient in the reference group was calculated. The fungal inhibition rate for each replicate well in the treatment group at different concentrations was determined using the following formula:

$$Inhibition\;Rate\;\left(\%\right)=\frac{\left(A_{NC}-OD_{T}\right)+\left(A_{Ref}-A_{B}\right)}{A_{NC}-A_{B}}\;\times 100$$

where *A_NC_* is the average OD value of the Negative Control group; *OD_T_* is the OD value of a single well in the Treatment group; *A_Ref_* is the average OD value of the Reference group at the corresponding concentration; *A_B_* is the average OD value of the Blank group.

### 4.4 Cell Wall Permeability Assay

#### 4.4.1 Calcofluor White Staining

2 tubes containing 500 µL culture of logarithmic‐phase fungi were centrifuged, washed with PBS, and recentrifuged. The pellets were resuspended in 500 µL CFS‐P (25 µg/mL) and PBS control, respectively, at 30°C for 12 h. After incubation, cells were collected by centrifugation, washed 1–2 times with PBS, and stained with 500 µL Calcofluor White (25 µm) working solution at 30°C in the dark for 10 min. After washing, cells were observed under a laser scanning confocal microscope (Ex: ∼336 nm, Em: ∼430 nm).

#### 4.4.2 Hoechst 33342 Staining

5 tubes containing 500 µL culture of logarithmic‐phase fungi were centrifuged, washed with PBS, and recentrifuged. The pellets were resuspended in 500 µL CFS‐P (6.25, 12.5, 25, 50 µg/mL) and PBS control, respectively, at 30°C for 8 h. After incubation, cells were collected, washed 1–2 times with PBS, and stained with 500 µL Hoechst 33342 (10 µg/mL) working solution at 30°C in the dark for 10 min. Unbound dye was removed by centrifugation and washing. Cells were resuspended in 1 mL PBS, and 200 µL of the suspension was placed on a confocal dish for observation of blue fluorescence intensity (Ex max = 350 nm, Em max = 461 nm).

#### 4.4.3 Rhodamine B staining (Concentration Gradient)

The cell pretreatment procedure was identical to that described for Hoechst 33342 staining. Following incubation with CFS‐P solutions (6.25, 12.5, 25, 50 µg/mL; PBS served as the control), the cells were stained with Rhodamine B (5 µg/mL) working solution for 30 min. Subsequently, the staining solution was removed by centrifugation, and the cells were washed with PBS. Finally, the cells were resuspended in a small volume of PBS and transferred to a confocal dish for observation (Rhodamine B: Ex max = 554 nm, Em max = 627 nm).

#### 4.4.4 NanoDrop One (Thermo Scientific) Measurement of Supernatant Protein/Nucleic Acid

The experiment included three groups, each with three replicates. *C. albicans* in the logarithmic phase was centrifuged, washed with PBS, and recentrifuged. The control group pellet was resuspended and incubated with PBS. The CFS‐P group pellet was resuspended and incubated with CFS‐P solution (25 µg/mL). The Mannan blocking group pellet was resuspended and incubated with a mixture containing CFS‐P (25 µg/mL) and mannan (250 µg/mL). Samples were incubated at 30°C, and aliquots were taken at 0.5, 2, 4, and 8 h. After incubation, samples were centrifuged at 3000 rpm for 5 min. The supernatant was collected, and the concentrations of protein and nucleic acids were measured using a NanoDrop instrument.

#### 4.4.5 ITC (Isothermal Titration Calorimetry)

Mannan was selected as the titrant due to its good water solubility, with a test concentration set at 4 mm, and this concentration was calculated with the molecular weight of the mannose monomer. The nanocrystals (CFS, CFS‐G, CFS‐I, and CFS‐P) served as the samples in the cell. Their concentrations were standardized based on copper content, with all sample concentrations adjusted to 0.3 mm for testing. For the experimental groups, mannan was titrated into samples of CFS, CFS‐G, CFS‐I, and CFS‐P, respectively. Control experiments involved titrating mannan into the corresponding blank solvent to correct for background thermal effects.

#### 4.4.6 ROS Scavenger Quenching Assay

Candida albicans cells in the logarithmic phase were diluted to 1 × 10^5^ CFU/mL. The ROS scavengers used were TEMPO (2 µm), catalase (CAT, 40 U/mL), and isopropanol (IPA, 0.1 mm), quenching H_2_O_2_, ·OH, and O_2_^−^·. For each group, 1.1 mL of fungal suspension was mixed with 500 µL of CFS‐P solution and 500 µL of the indicated scavenger solution in a total volume of 2 mL, yielding a final CFS‐P concentration of 25 µg/mL. In the blank and antimicrobial control groups, an equal volume of sterile PBS was added instead of the scavenger. Samples were incubated at 37°C, and aliquots were collected at 0, 1, 2, and 4 h. At each time point, 100 µL of suspension was spread onto YM agar plates in triplicate and incubated at 30°C. Colonies were counted on plates, and viable counts were expressed as CFU/mL. The killing efficiency of each scavenger‐treated group was compared with that of the CFS‐P group without scavenger at each time point.

### 4.5 Computational Methods

#### 4.5.1 Adsorption Energy Calculation of Mannan on Different Crystal Planes

All spin‐polarized density functional theory (DFT) calculations were carried out using the Vienna ab initio simulation package (VASP) [56, 57], employing the projector augmented‐wave (PAW) method. The exchange–correlation potential was treated with the Perdew–Burke–Ernzerhof (PBE) generalized gradient approximation (GGA) [58, 59], and long‐range van der Waals interactions were incorporated using the DFT‐D3 method [60]. A plane‐wave kinetic energy cutoff of 480 eV was adopted. The electronic energy and ionic forces were converged to within 10^−^^4^ eV, and the atoms' residual forces were declined to less than 0.02 eV/Å. A vacuum layer of 20 Å was introduced perpendicular to the slab to eliminate spurious interlayer interactions. Data analysis and visualization were performed utilizing the VASPKIT [61] code and VESTA [62] software, respectively. The adsorption energy Eads is given by the following formula:

$$\Delta E\mathrm{ads}\;=E_{A+B}\;-E_{A}-E_{B}$$

where E_A + B_ is the total energy of slab A absorbed with B, E_A_ for A slab energy, and E_B_ for B molecule energy.

#### 4.5.2 Surface Energy Reduction Calculation

All spin‐polarized density functional theory (DFT) calculations were performed using the Vienna ab initio simulation package (VASP) [56, 57], employing the projector augmented‐wave (PAW) method. The exchange–correlation potential was treated within the generalized gradient approximation (GGA) parameterized by Perdew [58, 59], Burke, and Ernzerhof (PBE), with the inclusion of Grimme's DFT‐D3 scheme for van der Waals corrections [60]. A plane‐wave kinetic energy cutoff of 480 eV was adopted. The electronic energy was converged to within 10^−^^5^ Ev and the atoms' residual forces were declined to less than 0.02 eV/Å. A vacuum layer of 20 Å was introduced perpendicular to the slab model to mitigate spurious interlayer interactions. Post‐processing for data analysis and visualization was conducted using the VASPKIT [61] and VESTA [62] codes, respectively. The surface energy is expressed by the following equation:

$$\;E_{\mathrm{surface}}=\frac{\left(E_{\mathrm{slab}}-NE_{\mathrm{bulk}}\right)}{2A}$$

where *E*_slab_ represent the total energy of the relaxed slab materials, *E*_bulk_ for the bulk energy pei unit, N for *E*_slab_ multiple of *E*_bulk_, and *A_slab_* for the slab materials’ total area.

#### 4.5.3 Molecular Dynamics Simulation of the Interaction Between Mannan and Different Crystal Facets of CuFeSe_2_

The molecular structure of mannan and the crystal structure of CuFeSe_2_ were retrieved from the PubChem and Materials Project databases, respectively. Low‐index (321), (332), (220), and (112) surfaces of CuFeSe_2_ were generated by cleaving the bulk crystal in Materials Studio. All molecular dynamics (MD) simulations were performed with the GROMACS package using the Amber99SB‐ILDN all‐atom force field under the NPT ensemble. Periodic boundary conditions were applied in all directions to model the chemisorption of glucomannan on the CuFeSe_2_ surfaces. Each system was simulated for 200 ns at a constant temperature of 300 K. The Particle Mesh Ewald (PME) method handled long‐range electrostatic interactions with a direct‐space cutoff of 1.25 nm. For van der Waals interactions, a cutoff‐switching function was applied between 1.25 and 1.35 nm. The linear constraint solving algorithm was used to constrain the bond lengths.

### 4.6 Evaluation of Vacuolar Destruction by CFS‐P

#### 4.6.1 CellTracker Blue CMAC Staining

3 tubes containing 500 µL culture of logarithmic‐phase fungi were centrifuged, washed with PBS, and recentrifuged. The pellets were resuspended in 500 µL CFS‐P (50, 100 µg/mL) and PBS control, respectively, at 30°C for 12 h. After incubation, cells were collected, washed 1–2 times with PBS, and stained with 500 µL CMAC (5 µm) working solution in the dark at 30°C for 4 h. Cells were washed twice with 1–2 mL PBS and observed under a confocal microscope. CMAC (blue fluorescence) was excited at 405 nm, and emission was collected at 420–490 nm.

#### 4.6.2 FM4‐64 Staining (Vacuolar Membrane)

Three tubes containing 500 µL culture of logarithmic‐phase fungi were centrifuged, washed with PBS, and recentrifuged. The pellets were resuspended in 500 µL CFS‐P (50, 100 µg/mL) and PBS control, respectively, at 30°C for 12 h. After incubation, cells were collected, washed 1–2 times with PBS, and stained with 0.5 mL FM4‐64 (10 µm) working solution at room temperature in the dark for 5–30 min. Cells were centrifuged (6000 rpm, 3–4 min), washed twice with PBS (standing for 5 min each time), and resuspended in PBS. Samples were observed under CLSM. FM4‐64 was excited at 555 nm, and emission was collected between 570 and 700 nm. Disruption of vacuolar membrane integrity was indicated by abnormal fluorescence distribution patterns.

#### 4.6.3 Fluo‐4 AM Staining (Intracellular Calcium)

Three tubes containing 500 µL culture of logarithmic‐phase fungi were centrifuged, washed with PBS, and recentrifuged. The pellets were resuspended in 500 µL CFS‐P (50, 100 µg/mL) and PBS control, respectively, at 30°C for 12 h. After incubation, cells were collected, washed 1–2 times with PBS, and stained with 200 µL Fluo‐4 AM (5 µm) working solution at 30°C in the dark for 30 min. After washing, cells were optionally incubated for another 20–30 min to ensure complete hydrolysis of Fluo‐4 AM to Fluo‐4. Fluorescence was detected by confocal microscopy (Ex: 488 nm, Em: 512–520 nm) to indicate changes in intracellular Ca^2^^+^ concentration.

#### 4.6.4 DCFH‐DA Staining (Reactive Oxygen Species)

Three tubes containing 500 µL culture of logarithmic‐phase fungi were centrifuged, washed with PBS, and recentrifuged. Add 1 mL of prepared CFS‐P solutions (0, 50, 100 µg/mL) containing DCFH‐DA dye (10 µm). At 30°C for 20 min. After incubation, cells were centrifuged, washed 1–2 times with PBS to remove unbound dye, resuspended in 1 mL PBS, and 200 µL was placed on a confocal dish for observation (Ex: 480 nm, Em: 510–540 nm).

#### 4.6.5 Rhodamine 123 Staining (Mitochondrial Membrane Potential)

Six tubes containing 500 µL culture of logarithmic‐phase fungi were centrifuged, washed with PBS, and recentrifuged. The pellets were resuspended in 500 µL CFS‐P (6.25, 12.5, 25, 50, 100 µg/mL) and PBS control, respectively, at 30°C for 12 h. After incubation, cells were collected, washed 1–2 times with PBS, and stained with 500 µL Rh123 (10 µm) working solution at 30°C in the dark for 30 min. Unbound dye was removed by centrifugation and washing. Cells were resuspended in 1 mL PBS, and 200 µL of the suspension was placed on a confocal dish for observation of green fluorescence intensity (Ex max = 507 nm, Em max = 529 nm).

#### 4.6.6 Tetramethylrhodamine, Ethyl Ester (TMRE) Staining

Six tubes containing 500 µL culture of logarithmic‐phase fungi were centrifuged, washed with PBS, and recentrifuged. The pellets were resuspended in 500 µL CFS‐P (6.25, 12.5, 25, 50, 100 µg/mL) and PBS control, respectively, at 30°C for 12 h. After incubation, cells were collected, washed 1–2 times with PBS, and stained with 500 µL TMRE (10 µm) working solution at 30°C in the dark for 30 min. Unbound dye was removed by centrifugation and washing. Cells were resuspended in 1 mL PBS, and 200 µL of the suspension was placed on a confocal dish for observation of red fluorescence intensity (Ex max = 550 nm, Em max = 575 nm).

#### 4.6.7 ATP Quantification

The intracellular ATP levels in Candida albicans were quantified using a commercial ATP assay kit (Solarbio, Beijing, China) based on UV spectrophotometry. Log‐phase fungal cells were incubated with CFS‐P (12.5 or 50 µg/mL) or without treatment (control). After 4‐h incubation, the fungi were harvested, and the ATP was extracted with the kit extraction buffer, followed by quantification according to the manufacturer.

#### 4.6.8 Fungal Transcriptome Sequencing Analysis

*C. albicans* was co‐incubated with CFS‐P, CFS, CFS‐I, or CFS‐G (each at 100 µg/mL) for 6 h, with a PBS‐treated group as the control. After treatment, fungal pellets were collected, immediately frozen in liquid nitrogen, and subsequently subjected to transcriptome sequencing. This process included total RNA extraction, library construction, sequencing, and basic bioinformatics analysis. Functional annotation and cluster analysis of differentially expressed genes were performed using the Gene Ontology (GO) and Kyoto Encyclopedia of Genes and Genomes (KEGG) databases.

### 4.7 In Vivo Antifungal Efficacy

#### 4.7.1 In Vivo Antifungal Evaluation in a Cutaneous Fungal Infection Model

Female Balb/c mice (6–8 weeks old, 20–23 g) were randomly divided into control (saline), CFS‐P, and AmB groups (*n* = 6 per group). Abrasive wounds were created using sandpaper. A cotton swab dipped in a *C. albicans* suspension (1 × 10^7^ CFU/mL) was applied to the wound, which was then covered with a cotton ball and a 3 M transparent dressing for 24 h. At 24 h post‐infection, the wounds were treated with saline, CFS‐P (100 µg/mL), or AmB (100 µg/mL). Approximately 200 µL of the respective solution was applied to each wound using a cotton swab, once every 24 h for 5 consecutive treatments. Wound conditions were photographed, and mouse body weights were recorded on days 1, 3, 5, and 7. At 4–5 h after drug administration on days 1 and 4, wound fungal burden was assessed in 5 mice per group by swabbing the wound with a PBS‐soaked cotton swab, vortexing the swab in 1 mL PBS, and plating 100 µL of the suspension (in triplicate). On days 4 and 7, one skin tissue sample per group was fixed in 4% paraformaldehyde, embedded in paraffin, sectioned, and subjected to H&E and Masson staining. On day 7, all mice were euthanized, and heart, liver, spleen, lung, and kidney samples were collected for H&E staining analysis.

#### 4.7.2 In Vivo Antifungal Evaluation in a Deep‐Skin Mycosis Infection Model

Female Balb/c mice (6–8 weeks old, 20–23 g) were divided into control (saline), AmB, and CFS‐P groups (*n* = 6 per group). After anesthesia and shaving of the back, all mice received an intradermal injection of 100 µL of *C. albicans* suspension (5×10^6^ CFU/mL). After edema formation, 50 µL of saline, CFS‐P (100 µg/mL), or AmB (100 µg/mL) was injected into the site. The first injection day was designated day 1, and interventions continued for 5 days. On Day 10, the mice were sacrificed, and the infected skin tissues were collected. From each group, five samples were homogenized in 1 mL of physiological saline containing 0.1% Triton X‐100. The homogenate was centrifuged at low speed (1000 rpm, 10 min), and the supernatant was plated on SDA plates. After incubation at 30°C for 48 h, colonies were counted to determine the average viable fungal load (CFU/g) in the wound skin. One additional skin sample per group was fixed in 4% paraformaldehyde overnight, embedded in paraffin, sectioned, and stained with H&E and Masson.

### 4.8 Statistical Analysis

Data were presented as mean ± SD, and one‐way analysis of variance was used for statistical analysis, in which *p< 0.05, **p< 0.01, ***p< 0.001, and ****p< 0.0001 were considered statistically significant. GraphPad Prism software was used.

## Author Contributions

Z.W., X.D., and X.H. contributed equally to this work. Z.W. was mainly responsible for conceptualization, supervision, writing, and funding acquisition. X.D. and X.H. contributed specific investigations, formal analysis, visualization, and methodology. Y.P. contributed methodology. Y.Q., L.Z., and L.X. contributed to the data analysis. T.Q. and D.Z. provided resources. Y.M. and Y.W. provided resources, supervision, and funding acquisition.

## Conflicts of Interest

The authors declare no conflicts of interest.

## Supporting information




**Supporting File**: advs75259‐sup‐0001‐SuppMat.docx.

## Data Availability

The data that support the findings of this study are available from the corresponding author upon reasonable request.
