# Supplementary material for: Crystal Facet‐Polysaccharide Matching in CFS‐P Nanocrystals Drives Fungal Uptake, Vacuole Destruction, and Selective Antifungal Activity
Source: Adv Sci (Weinh). 2026 Apr 20;13(39):e24219. doi: 10.1002/advs.202524219 (PMC13335081; doi:10.1002/advs.202524219)
Supplement: Supplementary file 1 — Supporting File: advs75259‐sup‐0001‐SuppMat.docx. [file ADVS-13-e24219-s001.docx]

Supporting Information

**Crystal Facet-Polysaccharide Matching in CFS-P Nanocrystals Drives Fungal Uptake, Vacuole Destruction, and Selective Antifungal Activity**

*Zhaohui Wang,* Xiaojin Deng, Xujuan Huang, Yuhang Pan, Yao Qin, Lu Zhang, Le Xu, Tianyi Qin, Deteng Zhang, Yi Ma,* Yalong Wang**

**Supplementary Text**

**S1. Synthesis of nanocrystals**

**Synthesis of nanocrystal CFS.** The procedure was identical to that for CFS-P, except that PVP was omitted from the metal salt solution.

**Synthesis of nanocrystal CFS-I.** The procedure was identical to that for CFS-P, except that polyethylenimine (PEI, 100 mg) was used instead of PVP in the metal salt solution.

**Synthesis of nanocrystal CFS-G.** The procedure was identical to that for CFS-P, except that Polyethylene glycol (Thiol PEG, 100 mg, 2,000 Da) was used instead of PVP in the metal salt solution.

**Synthesis of nanocrystal Fe-Se.** Selenium powder (21.8 mg) was weighed and placed into a dry three-necked flask. Then, 48 mL of ddH₂O was added, and a stir bar was placed inside. The system was purged with nitrogen for 30 minutes. Sodium borohydride (35 mg) dissolved in 1 mL of ddH₂O was injected along the wall using a 2.5 mL syringe. After reacting for 2 hours until clear and colorless, FeCl₂ (16.21 mg) and PVP (100 mg) were dissolved in 2 mL of ddH₂O (ultrasonicated for ~3 minutes) was rapidly injected into the system. The reaction proceeded for 1 hour. Completion was confirmed by clarity of a diluted aliquot. The sample was dialyzed (100,000 Da) against water for 48 hours (one water change), lyophilized, and stored.

**Synthesis of nanocrystal Cu-Se.** Selenium powder (39.48 mg) was placed in a 100 mL three-necked flask, followed by 48 mL of deionized water and a stir bar. The flask was sealed, stirred at 200 rpm, and purged with nitrogen for 30 minutes. Sodium borohydride (56.75 mg) was dissolved in 2 mL of deionized water, ultrasonicated for 30 seconds, and rapidly injected into the flask along the wall. The stirring speed was increased to 700 rpm, and the reaction proceeded at room temperature for 2 hours until the solution became clear and colorless. Finally, a mixture of anhydrous copper chloride (134 mg) and PVP (100 mg), dissolved in 2 mL of deionized water via sonication, was added to the reaction system. The reaction continued for 1 hour at 1250 rpm. The resulting suspension was collected, purified by dialysis, and the final solid product was obtained by freeze-drying.

**Synthesis of nanocrystal Cu-W-Se.** Selenium powder (39.48 mg) was accurately weighed into a three-necked flask containing 44 mL of deionized water. Nitrogen purging continued for 30 minutes. Then, 1.5 mL of a pre-prepared sodium borohydride solution (126.1 mg NaBH₄ in 2 mL water) was injected. Once the reaction mixture turned from turbid to clear and colorless, indicating complete reduction of selenium to selenide, a solution of tungsten hexachloride (49.57 mg WCl₆ in 2 mL anhydrous ethanol) and a solution of copper chloride (33.61 mg CuCl₂ in 2 mL water) were simultaneously injected into the flask using two syringes under continuous stirring and nitrogen atmosphere. Simultaneously, 2 mL of an aqueous solution containing PVP (140 mg, Mw = 8000) was added as a surfactant. The reaction proceeded at room temperature for 1 hour. The resulting suspension was collected, purified by dialysis, and lyophilized.

**Synthesis of nanocrystal Cu-Fe-S.** The synthesis of Cu-Fe-S nanocrystal was conducted under a nitrogen atmosphere. Na_2_S powder (7.81 mg) was added to a dry three-necked flask containing 48 mL of ddH₂O. After purging with N₂ for 30 minutes, an aqueous solution of NaBH₄ (35 mg in 1 mL ddH₂O) was injected. The reaction proceeded for 2 hours until a clear, colorless solution was obtained. Subsequently, a solution of CuCl₂ (6.72 mg), FeCl₂ (6.34 mg) and PVP (100 mg) in 2 mL ddH₂O was sonicated, rapidly injected into the flask, and reacted for 1 hour. Successful nanoparticle formation was confirmed by the clarity of a diluted aliquot. The product was purified by dialysis against water (100 kDa MWCO, 48 h), followed by lyophilization to obtain the final powder for storage.

**S2. In vitro fungicidal evaluation of CFS-P.**

**Plating assay.** *C. albicans*, *C. tropicalis*, and *C. glabrata* cultures in the logarithmic growth phase were diluted with YM medium to 10⁵ CFU/mL. For the negative control, 100 μL of each fungus suspension was mixed with 100 μL of PBS buffer in a tube. For the CFS-P group, CFS-P was diluted to 25 μg/mL in PBS, and 100 μL of this solution was mixed with 100 μL of the diluted bacterial suspension. The tubes were incubated statically at 30°C for 2-3 hours. Then, 100 μL from each tube was spread evenly on solid agar plates. The plates were incubated at 30°C until colonies formed, and images were captured. For *Aspergillus terreus*, the final CFS-P concentration incubated with fungi was 25 μg/mL, and Potato Liquid Medium with Chloroamphenicol and Intergrated Potato Medium were used for culturing.

**Crystal violet staining (Biofilm).** *C. albicans* suspension (1 mL, 10⁷ CFU/mL) was added to each well of a 96-well plate and incubated at 30°C for 48 hours to form biofilms (fresh medium was added if evaporation occurred). The supernatant was carefully removed. Then, 200 μL of either CFS-P (100 μg/mL), AmB (100 μg/mL), or PBS (control) was added to the respective wells. The plate was incubated at 30°C for 24 hours. After incubation, the supernatant was discarded, and the biofilms were gently washed with sterile PBS and dried. Biofilms were fixed with 100 μL of 2.5% glutaraldehyde for 15 minutes, washed with PBS, and dried. Staining was performed with 100 μL of 0.1% crystal violet for 30 minutes. Excess stain was removed, and the wells were dried. After thorough washing with PBS and drying, images of the stained biofilms were captured. For semi-quantification, 200 μL of ethanol was added to each well to dissolve the crystal violet, and the absorbance was measured at 590 nm.

**Biofilm staining with SYTO-9/PI**. *C. albicans* suspension (1 mL, 10⁷ CFU/mL) was added to two confocal dishes and incubated at 30°C for 48 hours for biofilm formation. The medium was gently aspirated, and 1 mL of PBS (control) or CFS-P solution (200 μg/mL) was added to each dish, followed by incubation at 30°C for 24 hours. After drug incubation, the solution was carefully removed. SYTO-9 and PI staining solutions were added, and the dishes were incubated at room temperature in the dark for 15 minutes. Observation was performed under a confocal microscope: SYTO-9 (live cells, green fluorescence) using the FITC channel (Ex/Em max: 485/530 nm), and PI (dead cells, red fluorescence) using the Cy3 channel (Ex/Em max: 485/630 nm).

**Suspended cell staining with SYTO-9/PI.** 2 tubes containing 1 mL of fungal suspension (10⁶ CFU/mL) were centrifuged (6000 rpm, 5 min), and the supernatant was discarded. The pellets were washed once with PBS and centrifuged again. Then, 1 mL of CFS-P solution (100 μg/mL) was added to the test tube, and PBS was added to the control tube. The tubes were incubated at 30°C for 12 hours. After incubation, the cells were collected by centrifugation (6000 rpm, 5 min), washed 1-2 times with PBS, and stained with 1 mL of SYTO-9/PI staining solution for 15 minutes at room temperature in the dark. Cells were observed under a confocal microscope using the FITC channel for SYTO-9 and the Cy3 channel for PI.

**S3. Characterization of nanocrystals**

**Scanning electron microscopy (SEM) imaging**. The conductive adhesive was attached to the sample stub. The nanocrystals CFS-P powder (or CFS, CFS-G, CFS-I powders) was evenly sprinkled onto the conductive adhesive. Subsequently, loosely adhered particles were removed using compressed air (or a rubber bulb). Samples with poor conductivity were sputter-coated with a thin layer of gold to enhance electron conduction. The samples were then loaded into the scanning electron microscope for observation and imaging.

**Transmission electron microscopy (TEM) Imaging.** A 100 μg/mL dispersion of the CFS-P (or CFS, CFS-G, CFS-I) nanocrystals was prepared and ultrasonicated for 1-3 minutes to disperse the nanocrystals. An ultrathin carbon film was used as the substrate. A 10 μL aliquot of the sample dispersion was dropped onto the carbon film and allowed to adsorb for 1 minute. After adsorption, the residual liquid was carefully blotted away using clean filter paper. The sample-loaded grid was then stored in an Eppendorf tube and placed in a desiccator for overnight drying prior to analysis. The samples were observed and imaged using transmission electron microscopy. Low-resolution imaging was performed to examine the general morphology, while High-Resolution TEM (HRTEM) was employed to resolve the crystal lattice fringes. Elemental distribution was assessed using EDS.

**FTIR (fourier transform infrared spectroscopy).** Approximately 10 mg of each sample (PVP, CFS-P) was thoroughly ground and homogenized. Fourier Transform Infrared (FTIR) spectra were subsequently acquired using an FTIR spectrometer.

**Raman Spectroscopy.** A minimal amount of the powder sample (PVP, CFS-P) was transferred onto a glass slide using a spatula and pressed into a smooth, flat surface. Raman spectra were then collected using a Raman spectrometer.

**UV-vis DRS (UV-visible diffuse reflectance spectra).** UV-visible diffuse reflectance spectra utilizes ultraviolet light to irradiate the sample (CFS, CFS-P) surface, ejecting photoelectrons. After obtaining the diffuse reflectance spectra of CFS and CFS-P, they are converted into absorption spectra. The band gap values, forbidden band widths, and other parameters of CFS and CFS-P are then determined through calculation.

**Hydrodynamic size and zeta potential of CFS-P.** A 50 μg/mL dispersion of CFS-P in ultrapure water was prepared. The hydrodynamic diameter and zeta potential of the nanocrystal in aqueous solution were measured using a Zeta potential and particle size analyzer. The dispersion was serially diluted until stable and reproducible measurements for both hydrated size and zeta potential were obtained.

**XRD (X-ray diffraction) for crystalline phase identification.** CFS, CFS-P, CFS-I or CFS-G powders were individually ground using an agate mortar until no visible particles remained, ensuring a homogeneous fine powder suitable for pelleting. The crystal structures were then characterized using an X-ray diffractometer.

**XPS (X-ray photoelectron spectroscopy).** Approximately 5 mg of the CFS, CFS-P, CFS-I or CFS-G powder sample was finely ground and adhered to a sample stub using double-sided conductive tape. The sample was then analyzed using an X-ray photoelectron spectrometer (XPS). The raw data were processed with Origin software, and chemical states of the elements were determined following peak deconvolution and fitting.

**S4. Evaluation of metabolic and oxidative stress responses**

**Pyruvate dehydrogenase (PDH) activity assay.** 3 tubes containing 500 μL of logarithmic-phase *C. albicans* culture were centrifuged, washed with PBS, and the pellets were resuspended in 1 mL of 50, 100 μg/mL of CFS-P solution (PBS as control) and incubated at 30°C for 24 hours. After incubation, cells were centrifuged at 6000 rpm for 3 minutes, and the supernatant was discarded. Then, 1 mL of Reagent 1 and 10 μL of Reagent 2 were added to the pellet. Cells were sonicated on ice (power 200W, ultrasound 3s, interval 7s, total duration 5 minutes) to disrupt them. The lysate was centrifuged at 4°C, 11000 g for 10 minutes, and the supernatant was kept on ice for assay. The microplate reader was preheated for 30 minutes, and the wavelength was set to 605 nm. A working solution was prepared (enough for 180 μL per sample, triplicates per sample, total 2.7 mL). For the blank well, 180 μL working solution and 10 μL water were mixed, and the absorbance at 605 nm was measured immediately ($A_{1}$) and after 1 minute 10 seconds ($A_{2}$). $\Delta A_{blank}=(A_{1}-A_{2})$ For the test wells, 180 μL working solution and 10 μL of the sample supernatant were mixed, and $A_{3}$ and $A_{4}$ were recorded similarly. $\Delta A_{test}=(A_{3}-A_{4})$. PDH activity (U/10⁴ cells) was calculated as:

$$PDH activity (U/{10}^{4} cells)=\frac{(\Delta A_{assay}-\Delta A_{blank})\times V_{total}\times{10}^{9}}{\varepsilon\times d\times V_{sample}\times500}=3.655\times(\Delta A_{assay}-\Delta A_{blank})$$

where $V_{total}$ is the total volume of reaction system, 1.9×10^-4^ L. $\varepsilon$ is the molar extinction coefficient of 2,6-dichloroindophenol, 2.1×10^4^ L/mol/cm. $d$ is the light path length of 96-well plate, 0.5 cm. the total number of cells (in units of 10⁴), and $F$ is the sample dilution factor.

**Malondialdehyde (MDA) content assay.** 3 tubes containing 1 mL of logarithmic-phase *C. albicans* culture were centrifuged, washed with PBS, and the pellets were resuspended in 1 mL of 50, 100 μg/mL of CFS-P solution (PBS as control) and incubated at 30°C for 24 hours. After incubation, cells were centrifuged, and the supernatant was discarded. Then, 2 mL of extraction buffer was added to the pellet, and cells were sonicated on ice (power 200W, ultrasound 3s, interval 10s, 30 cycles). The homogenate was centrifuged at 4°C, 8000 g for 10 minutes, and the supernatant was collected and kept on ice. The spectrophotometer/microplate reader was preheated for >30 minutes and zeroed with distilled water. For the test tube, 300 μL MDA detection working solution and 100 μL sample were mixed; for the blank tube, 300 μL working solution and 100 μL distilled water were used. Then, 100 μL of Reagent 3 was added to both tubes. The mixtures were heated in a 100°C water bath for 60 minutes (caution against bursting), cooled rapidly on ice, and centrifuged at 10000 g for 10 minutes at room temperature. Then, 200 μL of the supernatant was transferred to a 96-well plate, and the absorbance at 532 nm and 600 nm was measured.

$$\Delta A=(A532 test -A532 blank )-( A 600 test - A 600 blank )$$

MDA content (nmol/10⁴ cells) was calculated as:

$$MDA content (nmol/{10}^{4} cells)=\frac{\Delta A\times V_{total}}{\varepsilon\times d}\times{10}^{9}\times\frac{F}{N\times V_{sample}\times V_{extract}}=53.763\times\frac{\Delta A}{N}\times F$$

Where $V_{total}$ is the Total volume of reaction system, 5 $\times$ 10^-4^ L. $\varepsilon$ is the MDA molar extinction coefficient, 1.55 $\times$ 10^5^ L/mol/cm. $d$ is the Light path length of 96-well plate, 0.6 cm. $V_{sample}$ is the sample volume added, 0.1 mL. $V_{extract}$ is the volume of extract added, 1 mL. $N$ is the total number of cells (in units of 10⁴), and $F$ is the sample dilution factor.

**S5. Biocompatibility evaluation**

**In vitro cytotoxicity evaluation.** Cell viability was assessed using the CCK-8 assay. Cells from confluent dishes were trypsinized, counted, and resuspended. 100 μL of cell suspension containing 8,000 cells was added to each well of a 96-well plate. After overnight incubation until 70-80% confluency, the medium was aspirated. Then, 100 μL of CFS-P solutions at concentrations of 25, 12.5, 6.25, 3.125, 1.56, and 0 μg/mL were added to the wells (n=5 replicates per concentration). After 12 hours of incubation, the drug (CFS-P or AmB) containing medium was removed, and the wells were washed with 200 μL PBS. Subsequently, 100 μL of medium containing 10% CCK-8 reagent was added to each well. Following incubation, the absorbance at 450 nm was measured.

$$Cell Viability (\%)=\frac{A_{1}-B_{0}}{A_{0}-B_{0}}\times100$$

where $A_{1}$ is the average OD value of the absorbance of wells with cells, CCK-8, and drug; $B_{0}$ is the average OD value of the absorbance of wells with medium and CCK-8 but no cells; and $A_{0}$ is the average OD value of the absorbance of wells with cells and CCK-8 but no drug.

**Hemolysis assay.** Mouse orbital blood (500 μL) was collected and mixed with 5 mL of ultrapure water (10x blood volume). After centrifugation at 4°C, 3,000 rpm for 5 minutes, the supernatant was discarded. The pellet was resuspended in ultrapure water to 10 mL, mixed, and centrifuged at 10,000 g for 5 minutes. This washing step was repeated until the supernatant was clear. Finally, the red blood cells (RBCs) were resuspended in 10 mL of ultrapure water. In a 96-well microtiter plate, negative control (100 μL PBS + 100 μL RBC suspension), positive control (100 μL 2% Triton X-100 + 100 μL RBC suspension), and treatment groups (eight concentrations of CFS-P, 100 μL each + 100 μL RBC suspension) were set up in quintuplicate. The final CFS-P (or AmB) concentrations were 100, 50, 25, 12.5, 6.25, 3.12, 1.56, and 0 μg/mL. The plate was incubated statically at 37°C in a 5% CO₂ incubator for 1 hour. Then, it was centrifuged at 3,000 rpm for 25 minutes. The supernatant (100 μL) from each well was transferred to a new 96-well plate, and the absorbance at 540 nm was measured. The experiment was repeated three times.

$$Hemolysis rate (\%)=\frac{A_{1}-B_{0}}{A_{0}-B_{0}}\times100$$

$A_{1}$ is the average OD value of the absorbance of wells with Treatmen; $B_{0}$ is the average OD value of the absorbance of wells with Negative Control; and $A_{0}$ is the average OD value of the absorbance of wells with Positive Control.

**Calcein AM/PI staining (Mammalian cells).** NIH-3T3 cells (1×10⁶) were seeded into a 12-well plate (2 mL medium/well) and cultured overnight at 37°C. Upon reaching 80% confluency, the medium was replaced with fresh medium containing different concentrations of CFS-P (1.56, 6.25, 25 μg/mL) or AmB (same concentrations), with a medium-only control group. Incubation continued for 12 hours at 37°C. After incubation, the medium was aspirated, and cells were washed 2-3 times with 1× Assay Buffer. Then, 500 μL of 4 μM Calcein AM working solution was added to each well, mixed gently, and incubated at 37°C in the dark for 20 minutes. After washing once with PBS, 500 μL of 4.5 μM Propidium Iodide (PI) working solution was added, and incubation proceeded at 37°C in the dark for 5 minutes. Following a final PBS wash, 100-200 μL of PBS was added to keep cells moist. Cells were observed under a confocal microscope: live cells (green, Calcein AM, Ex/Em = 494/517 nm), dead cells (red, PI, Ex/Em = 535/617 nm).

**Acute toxicity model.** Female ICR mice (4-6 weeks old, 23-25 g) were divided into three groups (n=4 per group). The mice received tail vein injections of 100 μL of either CFS-P (2.5 mg/kg), CFS-P (10 mg/kg), or an equal volume of physiological saline (control). The injection day was designated Day 1. On Day 3, mice were euthanized, and whole blood, serum, and tissue samples (heart, liver, spleen, lung, kidney) were collected. Hemolysis was avoided during blood collection. Serum was obtained using coagulant tubes or sterile, nuclease-free EP tubes: whole blood was allowed to clot at room temperature for 2 hours, centrifuged at 2-8°C, 3000 rpm for 15 minutes, and the supernatant (serum) was stored. Whole blood was analyzed for routine hematological parameters. Serum was analyzed for toxicity-related biochemical indicators: Alanine Aminotransferase (ALT), Aspartate Aminotransferase (AST), Blood Urea Nitrogen (BUN), Creatinine (CREA), potassium, and sodium ions. Liver tissue was analyzed for Copper (Cu) content by ICP-OES. Heart, liver, spleen, lung, and kidney tissues were subjected to H&E staining and analysis.

**Figure S1 to S21**

**
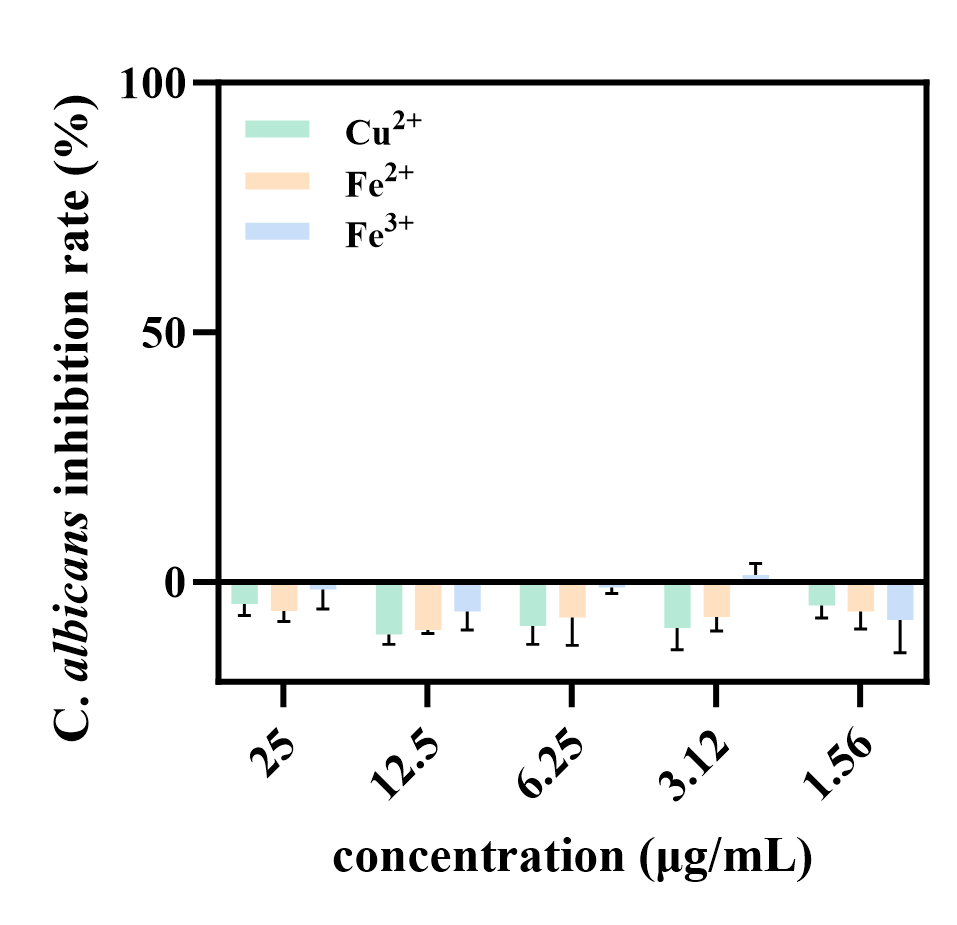
**

**Figure. S1.** Antibacterial activity of Cu^2+^, Fe^2+^, and Fe^3+^ against *C. albicans* at different concentrations. (Data were presented as mean ± sd, n=3)

**
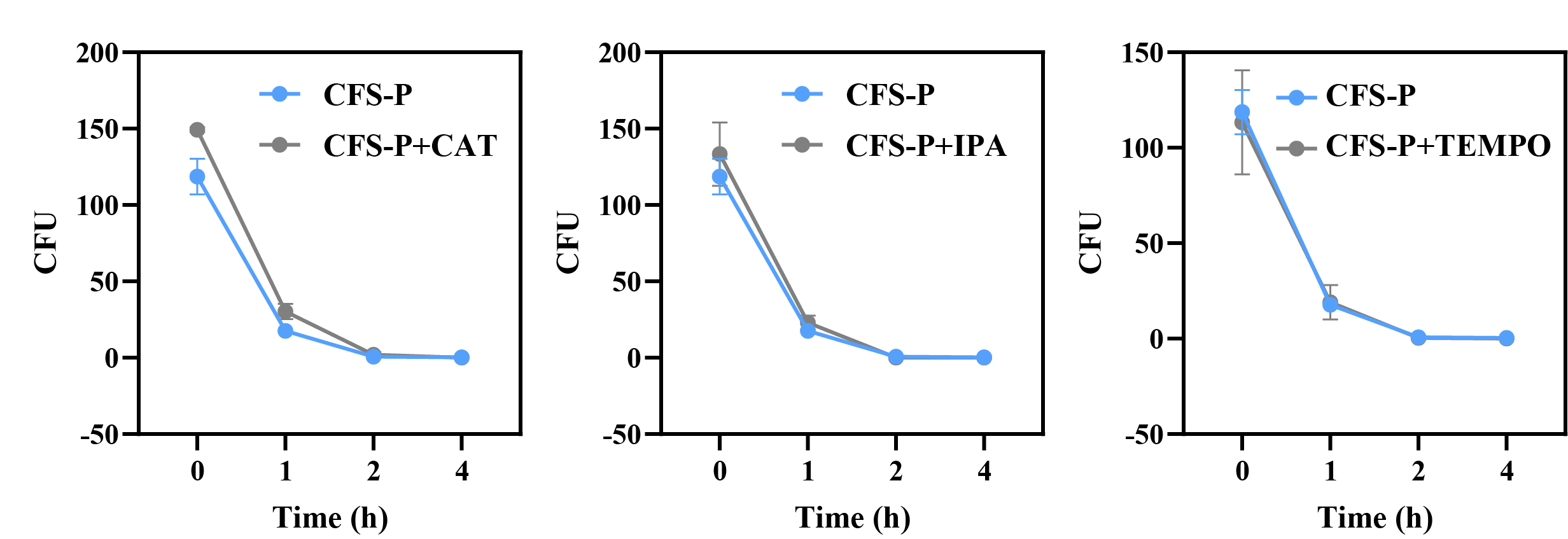
**

**Figure. S2.** Inhibitory rates of CFS-P and three ROS scavengers (CAT, IPA, and TEMPO) under different times (0, 1, 2, and 4 hours) against *C. albicans*. (Data were presented as mean ± sd, n=3) (Data were presented as mean ± sd, n=3)


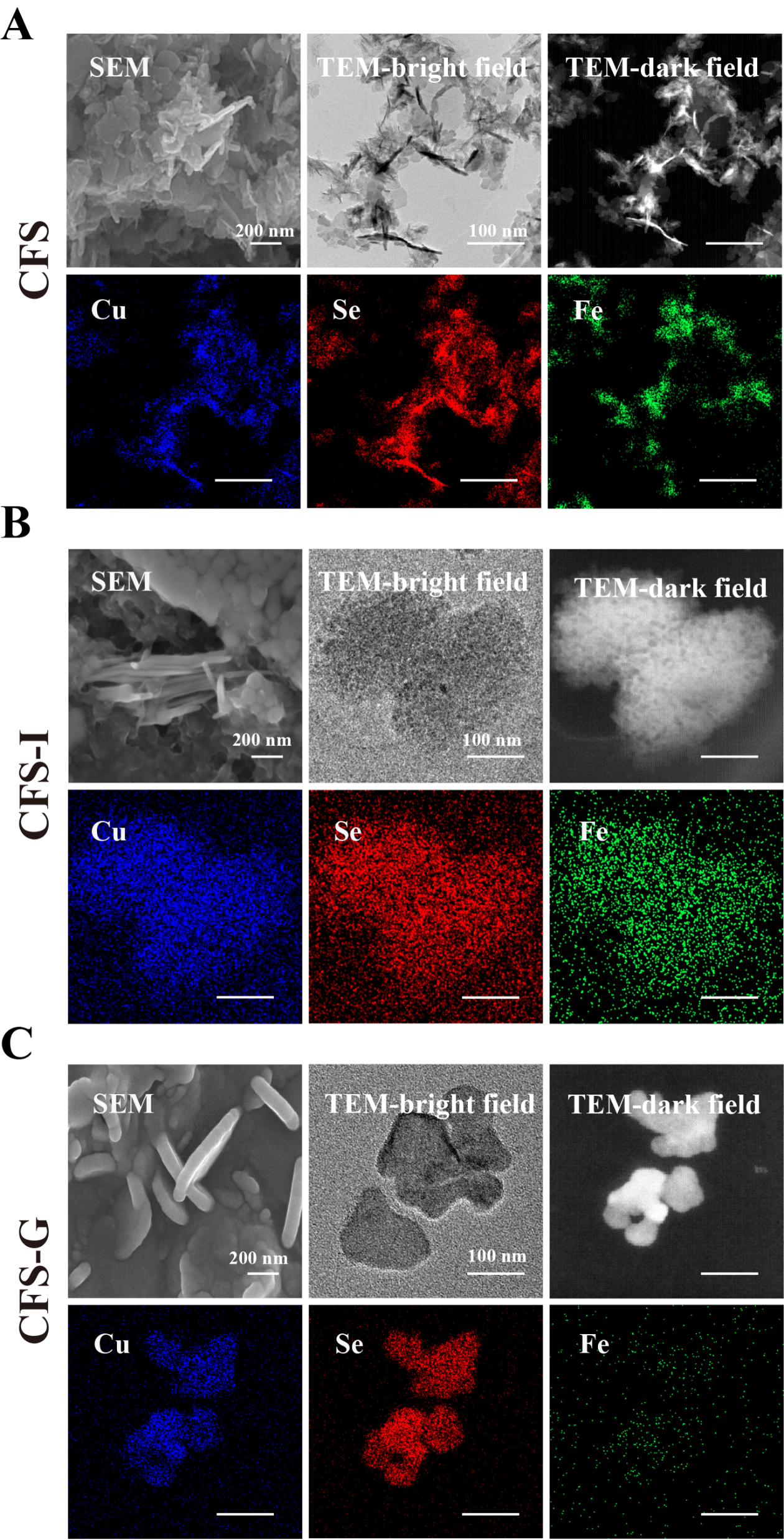


**Figure. S3.** Morphology of nanocrystals with different ligands under scanning electron microscopy and transmission electron microscopy, as well as elemental distribution analysis of Cu, Fe, and Se.


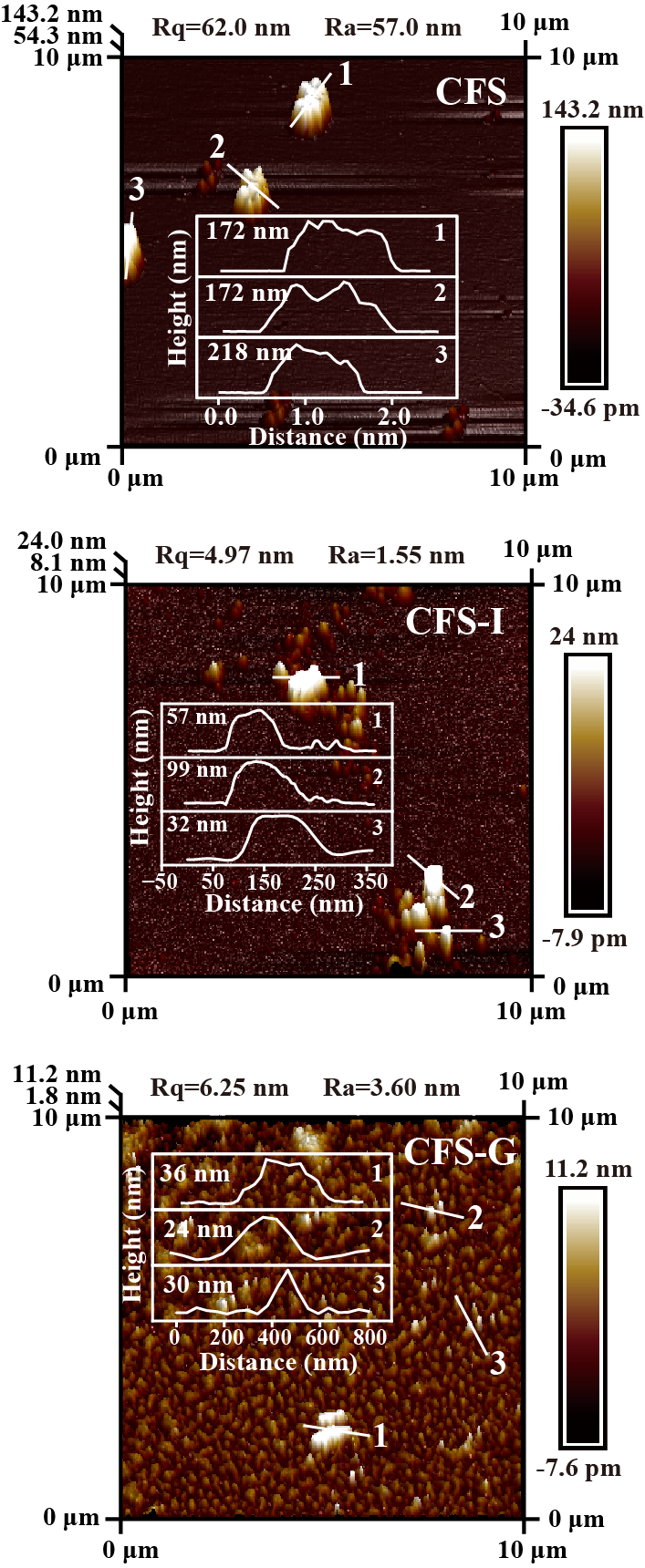


**Figure. S4.** Atomic Force Microscopy (AFM) images showing the thickness and roughness measurements of different nanocrystals.

**
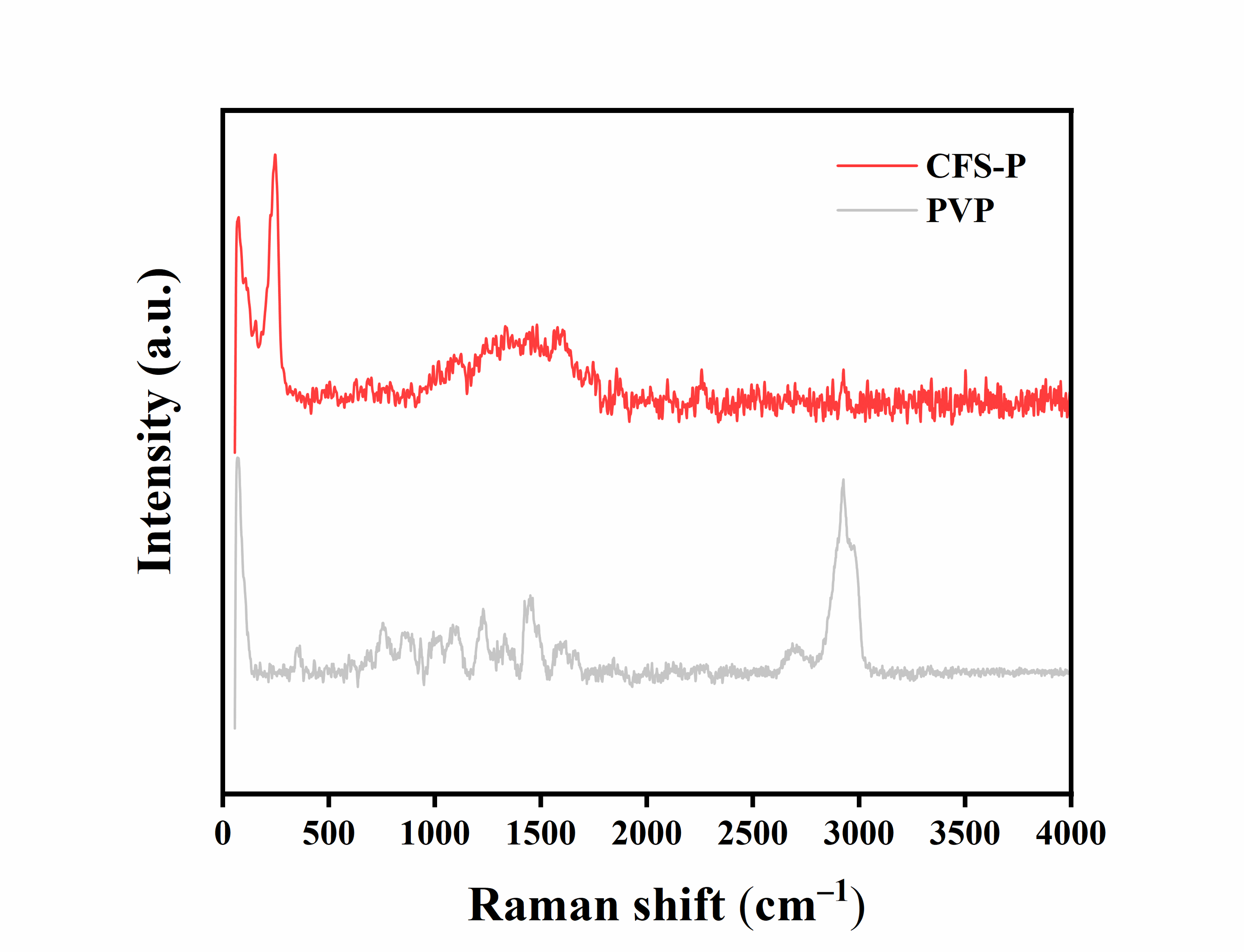
**

**Figure. S5.** Raman spectra of CFS-P and PVP.


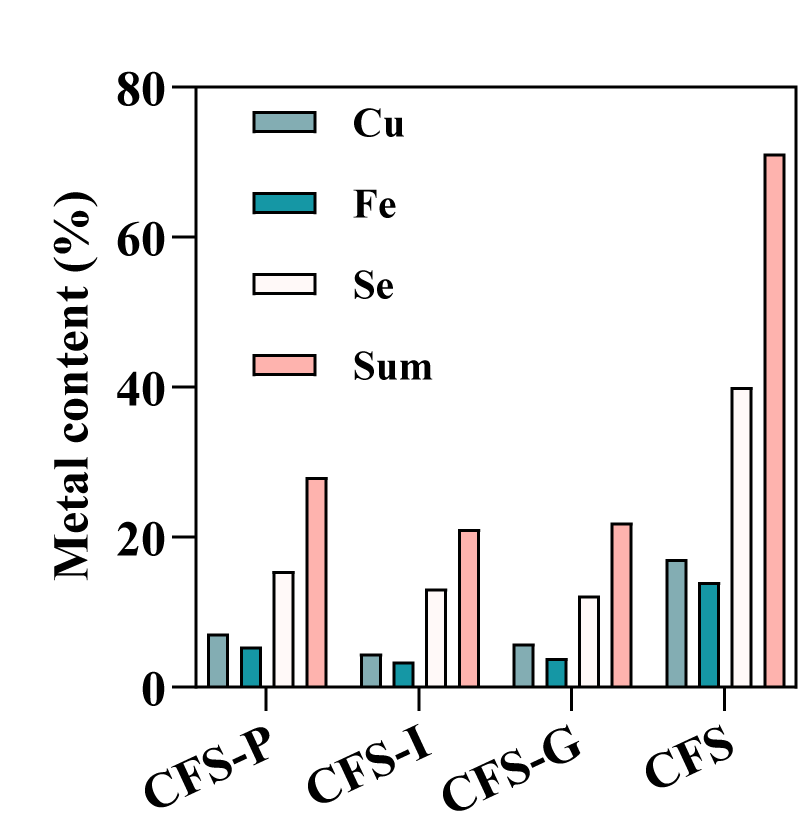


**Figure. S6.** Metal content of different nanocrystals (CFS-P, CFS-I, CFS-G, CFS) at the same mass concentration.


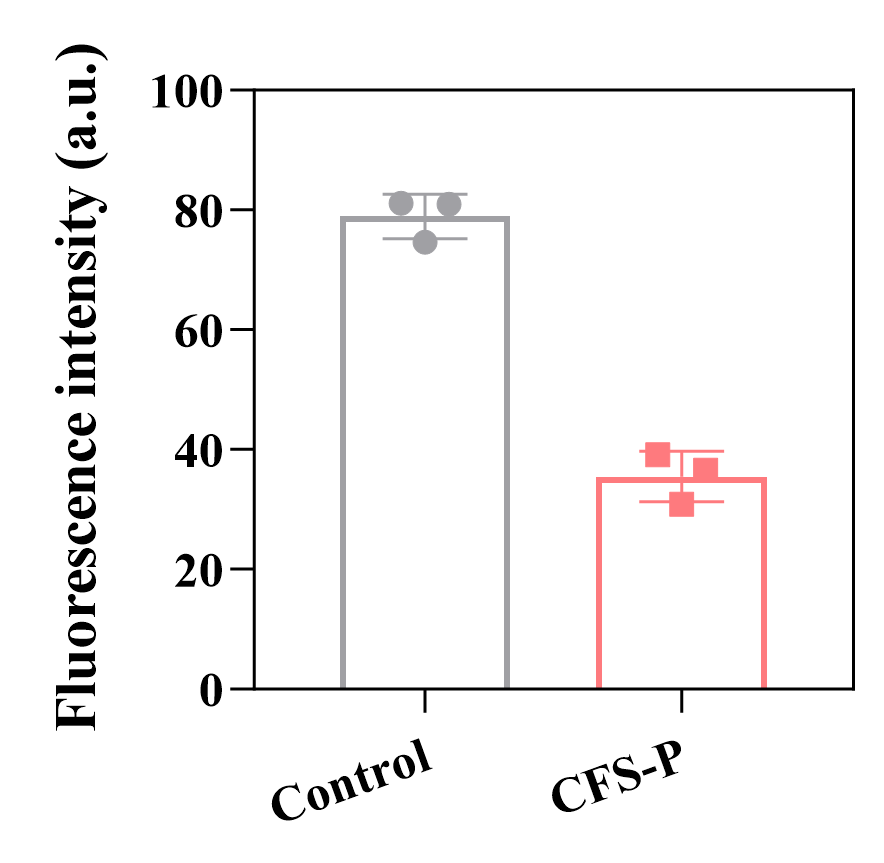


**Figure. S7.** Quantitative analysis of fluorescence images of *C. albicans* co-incubated with PBS or CFS-P and stained with Calcofluor White. (Data were presented as mean ± sd, n=3)


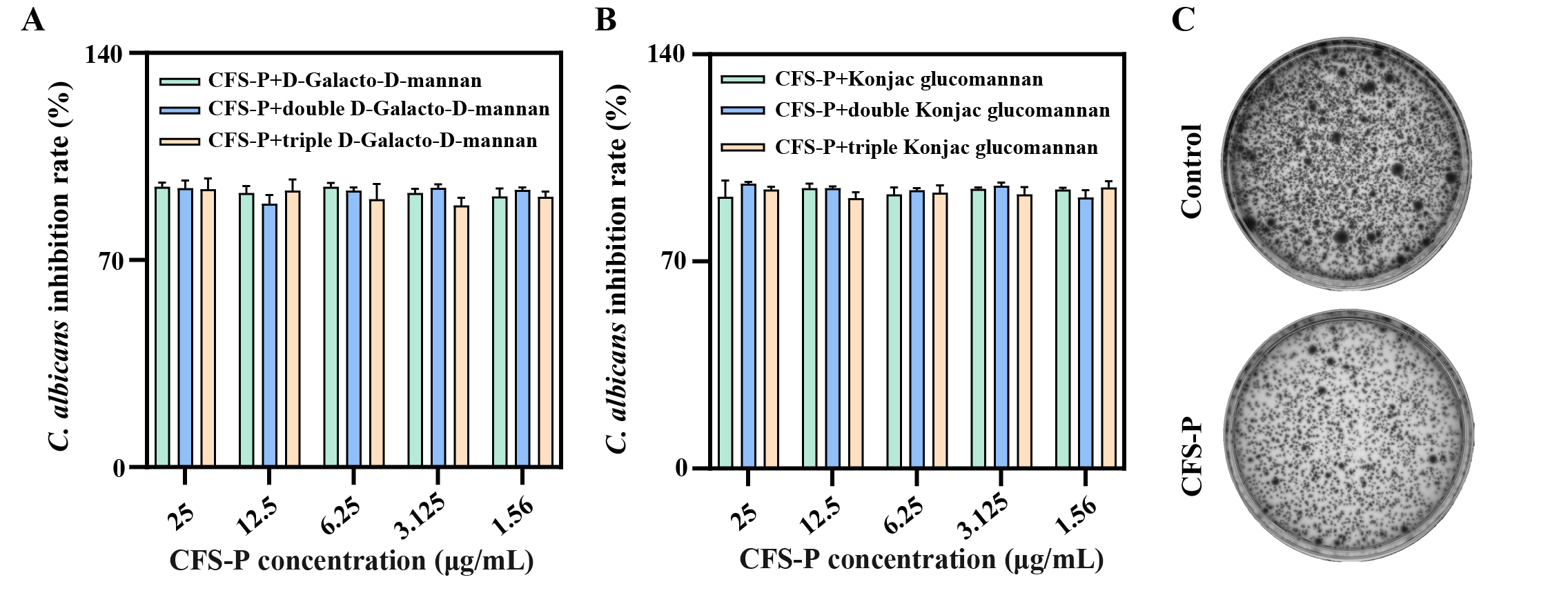


**Figure. S8.** (A, B) Inhibitory rates of CFS-P against *C. albicans* after pre-incubation of CFS-P with D-Galacto-D-mannan and Konjac glucomannan one-fold, two-fold, and three-fold of CFS-P concentrations. (Data were presented as mean ± sd, n=3) (C) Plating images of the inhibitory effects of CFS-P (25μg/mL) or control against *Aspergillus terreus.* (Data were presented as mean ± sd, n=3)


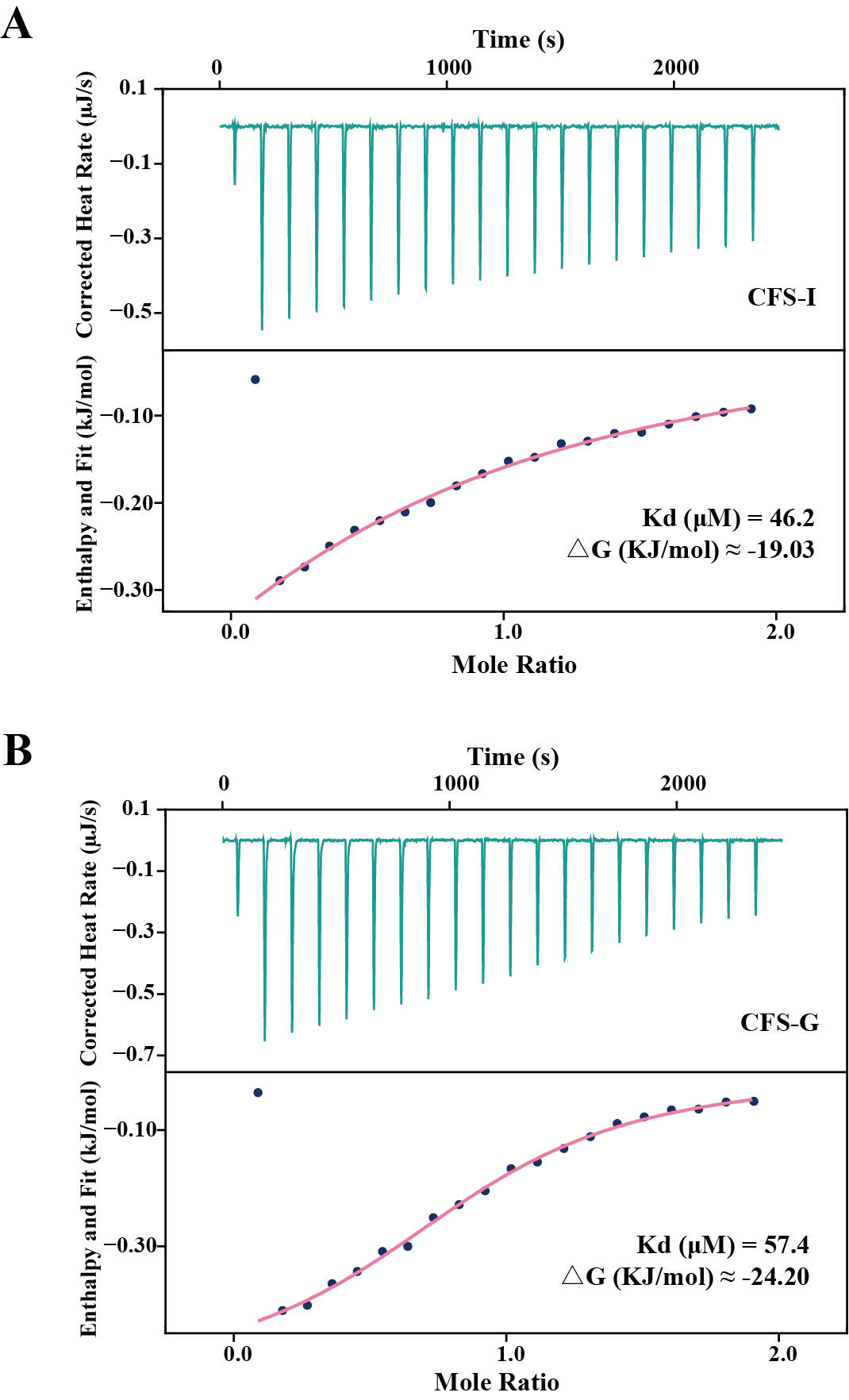


**Figure. S9.** Isothermal titration calorimetry (ITC) profiles of the binding of CFS-I (A) and CFS-G (B) with mannan.


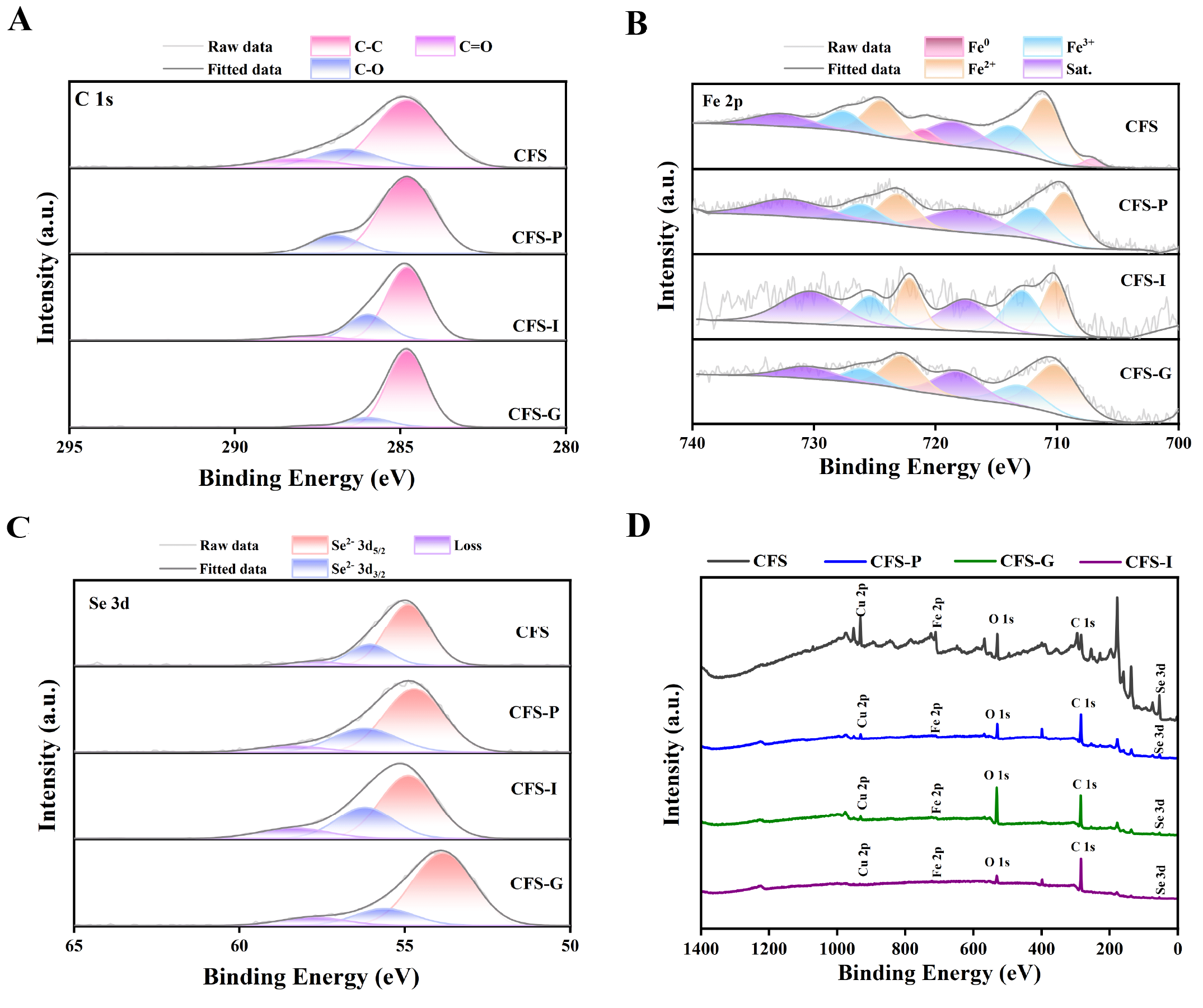


**Figure. S10.** X-ray photoelectron spectroscopy (XPS) spectra of different nanocrystals (CFS, CFS-P, CFS-I, CFS-G).


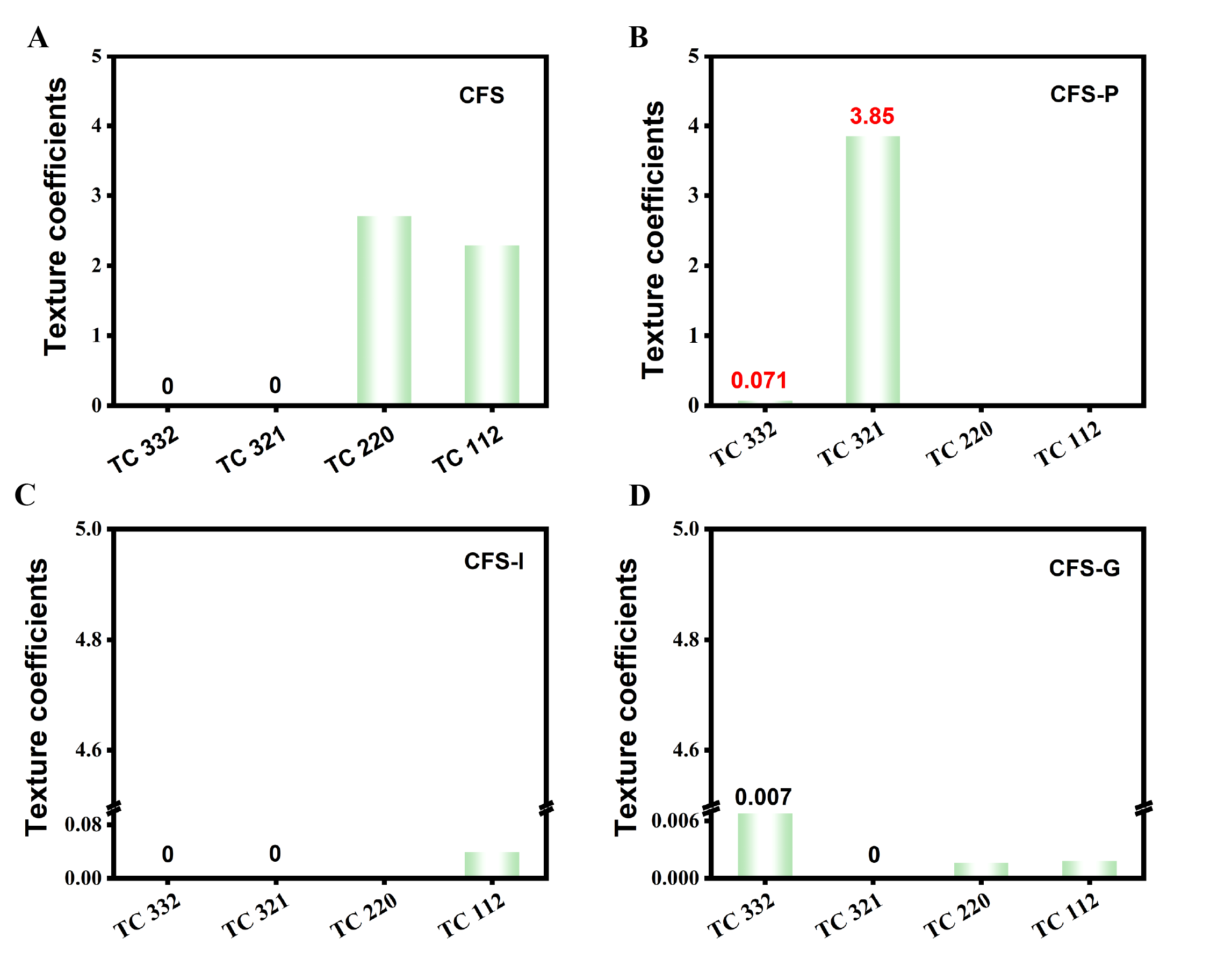


**Figure. S11.** Quantitative analysis based on X-ray diffraction (XRD) data to calculate the texture coefficients (TC) of CFS, CFS-P, CFS-I, and CFS-G.


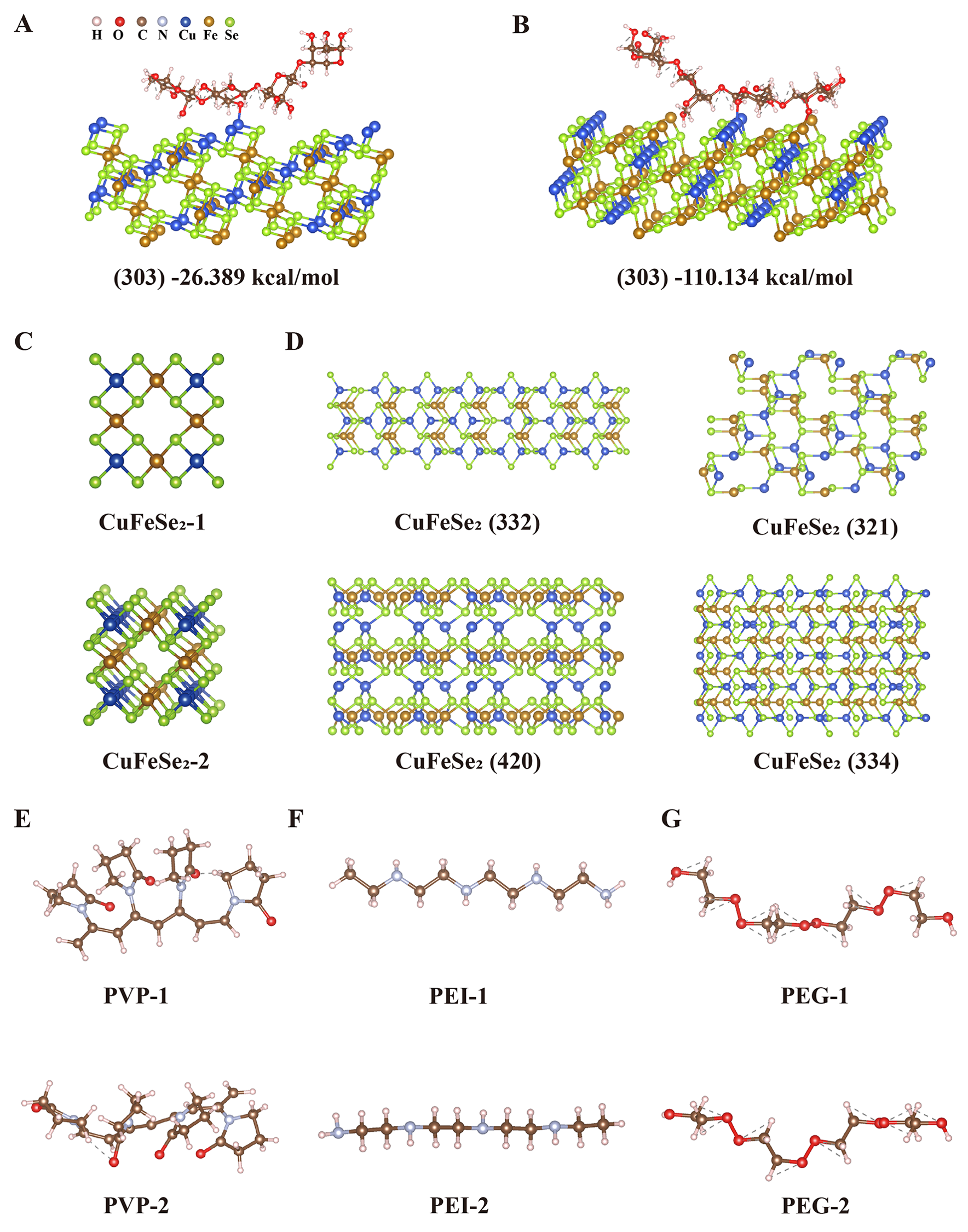


**Figure. S12.** Molecular dynamics simulation diagrams. (A) CuFeSe₂. (B) Various configurations of CuFeSe₂. (C-E) Ligand effects (PVP, PEI, PEG). (F, G) Configurations of CuFeSe₂ upon binding with PVP.


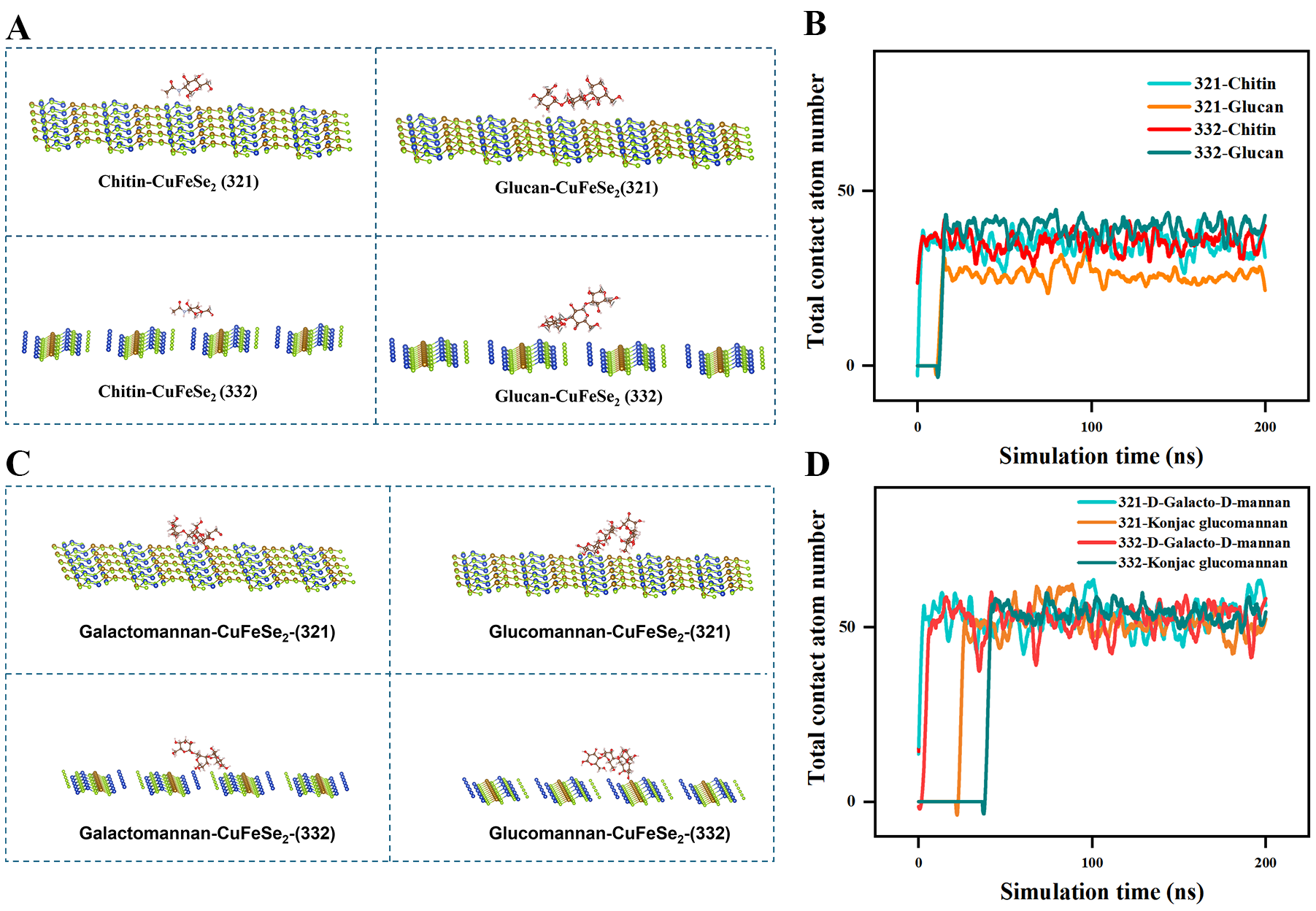


**Figure. S13.** (A) Molecular dynamics simulation snapshots of different crystal planes of CuFeSe₂ and two polysaccharides (chitin, glucan). (B) Number of chitin and glucan atoms bound to different crystal planes of CuFeSe₂. (C) Molecular dynamics simulation snapshots of different crystal planes of CuFeSe₂ and two polysaccharides (galactomannan, glucomannan). (D) Number of galactomannan and glucomannan atoms bound to different crystal planes of CuFeSe₂.

**
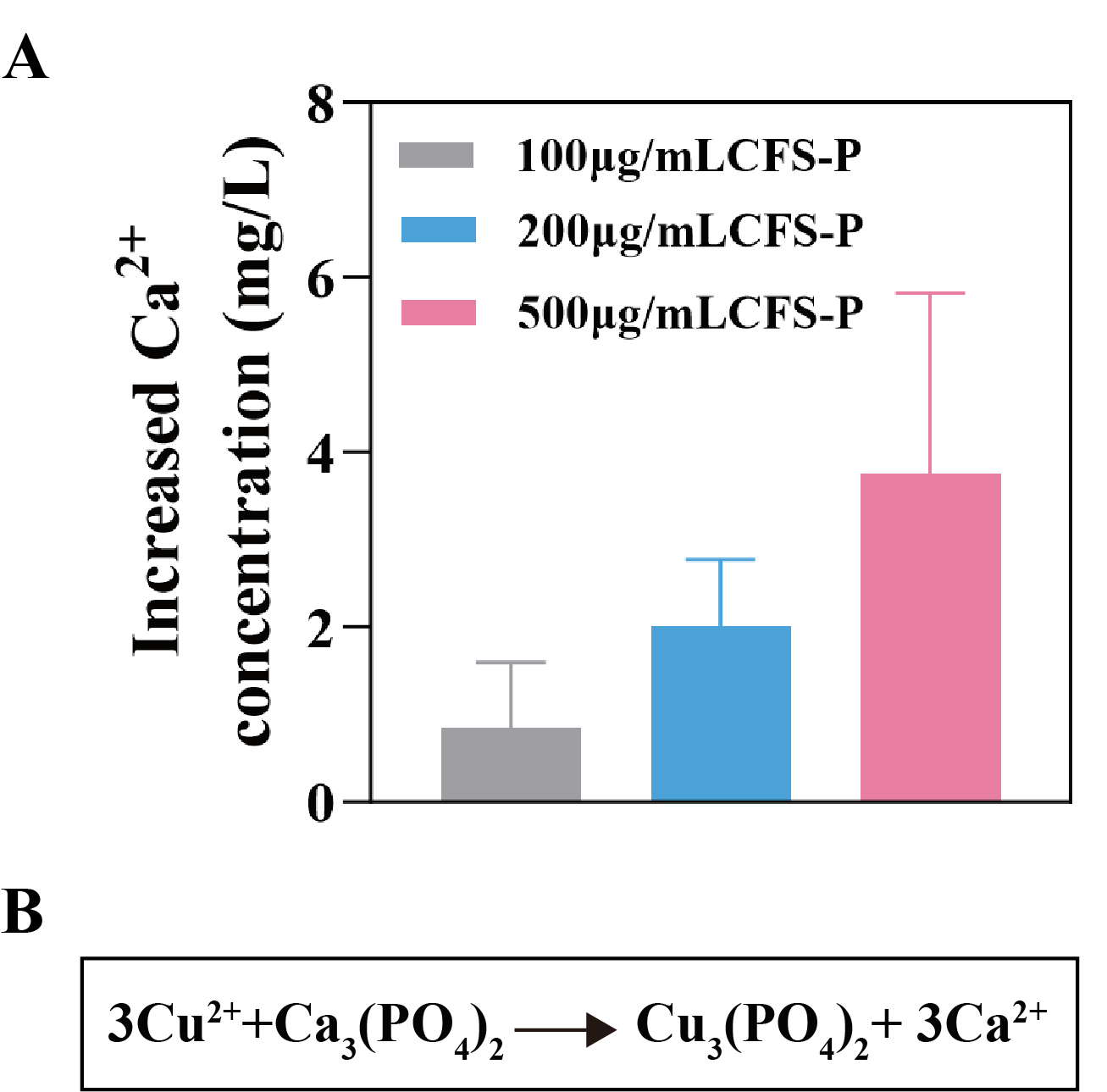
**

**Figure. S14.** ICP-OES analysis of changes in intracellular Ca²⁺ content in *C. albicans* following treatment with varying concentrations (100, 200, 500 μg/mL) of CFS-P. (Data were presented as mean ± sd, n=3)


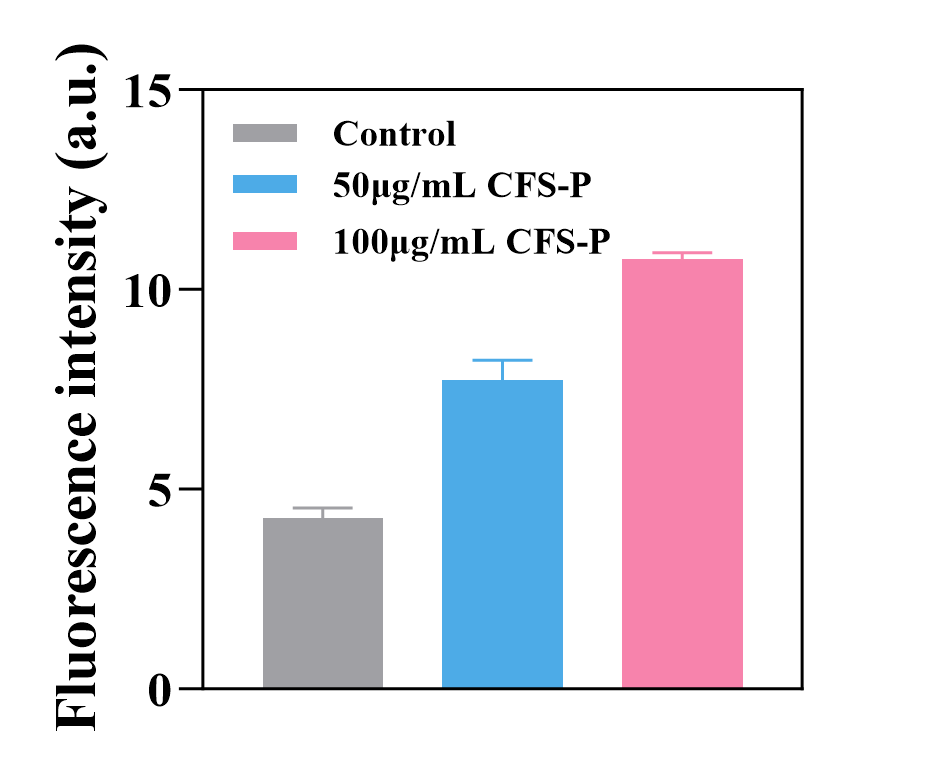


**Figure. S15.** Quantitative fluorescence analysis of DCFH-DA staining in *C. albicans* following co-incubation with varying concentrations (50, 100 μg/mL) of CFS-P or control. (Data were presented as mean ± sd, n=3)


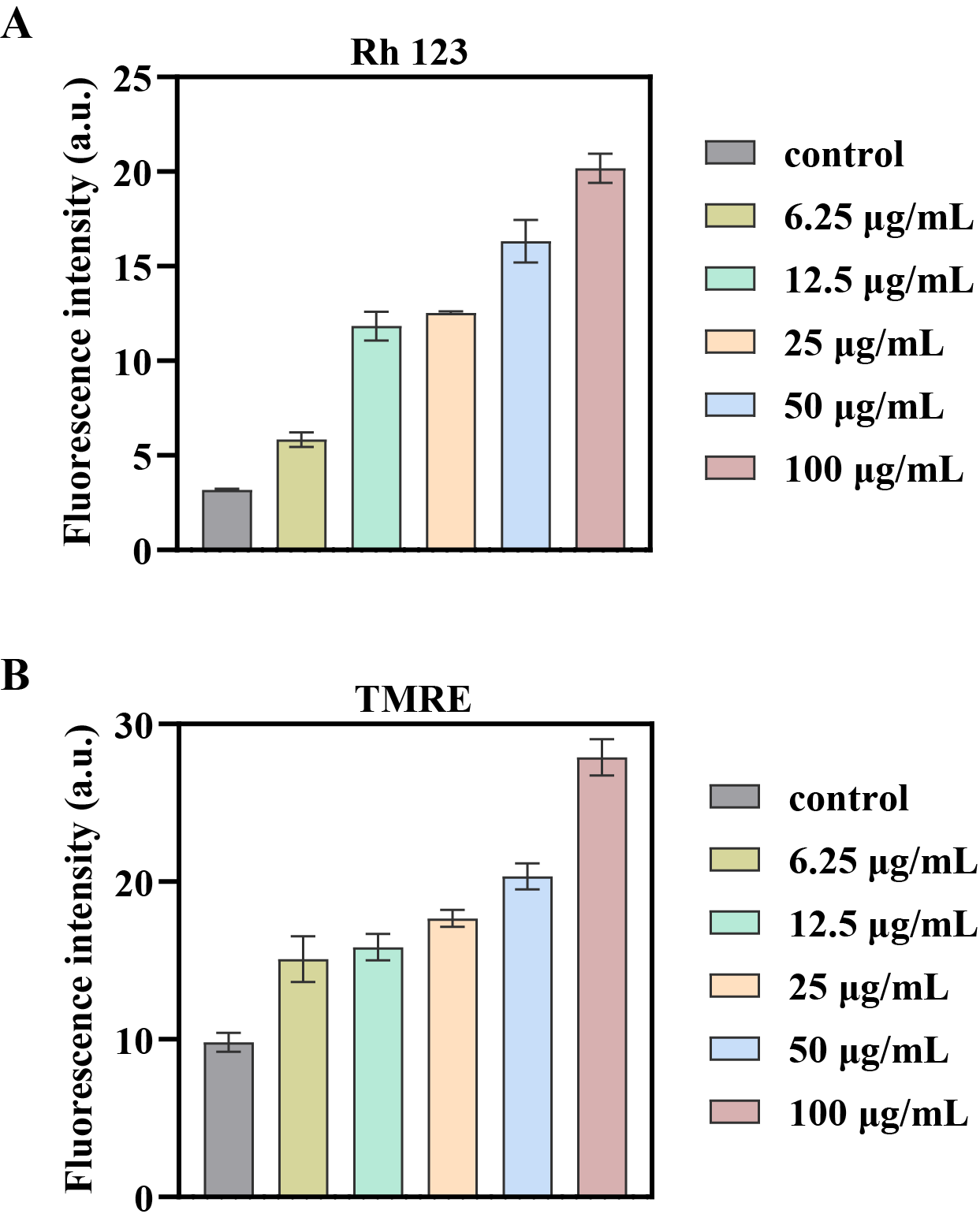


**Figure. S16.** Quantitative fluorescence analysis of Rhodamine 123 staining (A) or TMRE staining (B) in *C. albicans* following co-incubation with varying concentrations (6.25, 12.5, 25, 50, 100 μg/mL) of CFS-P or PBS (control). (Data were presented as mean ± sd, n=3)


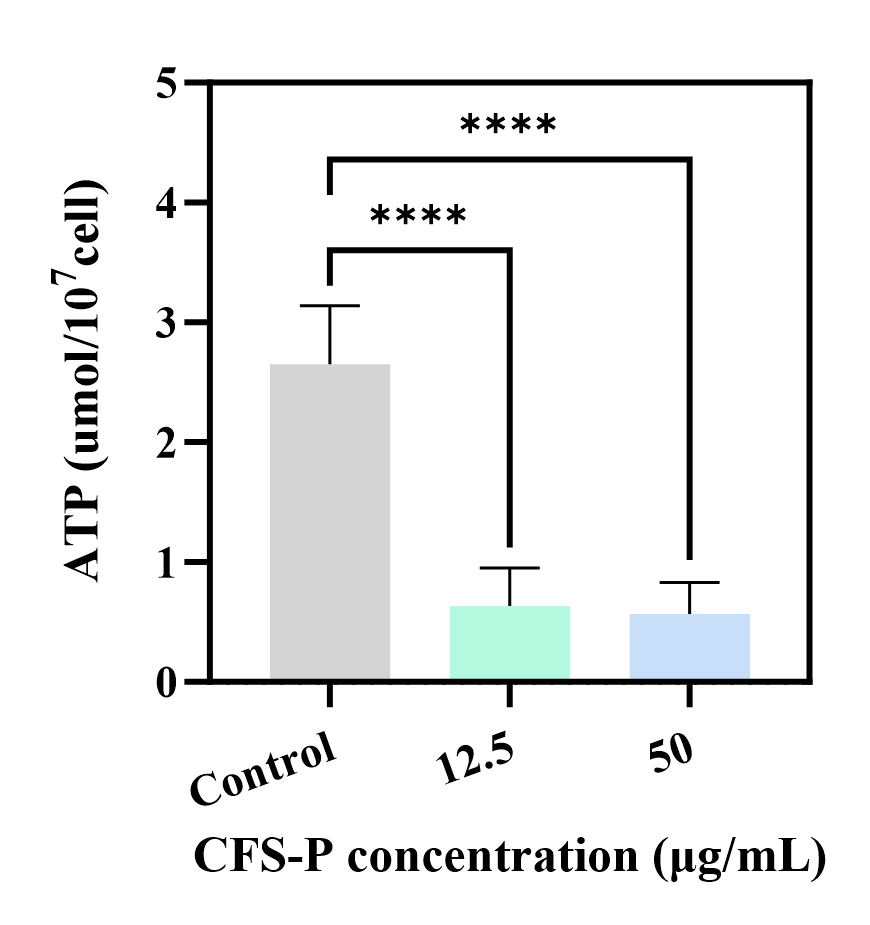


**Figure. S17.** Changes in ATP content of *C. albicans* under treatment with 12.5, 50 μg/mL CFS-P or PBS (control). (Data were presented as mean ± sd, n=6)


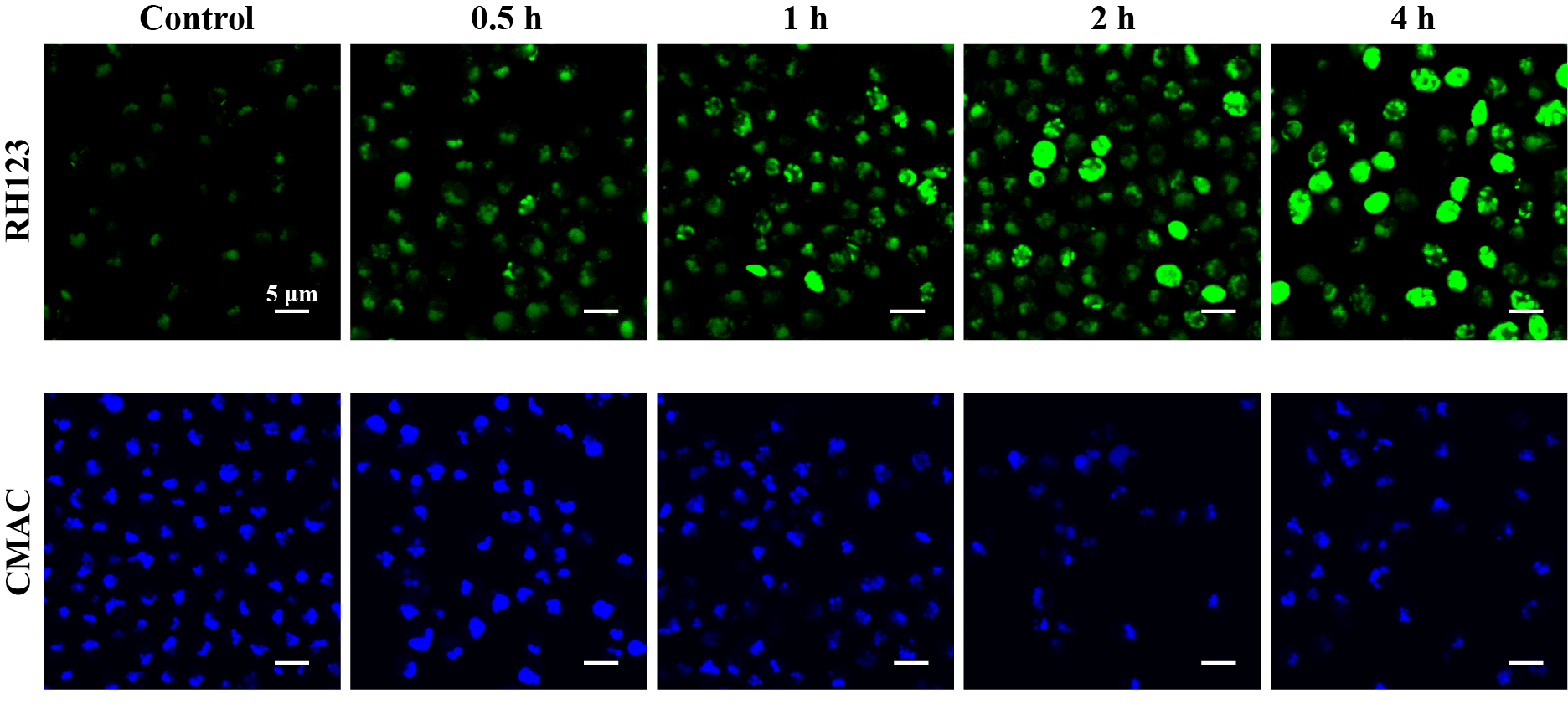


**Figure. S18.** Fluorescence images of *C. albicans* incubated with PBS or CFS-P and stained with Rhodamine 123 or CMAC dyes.


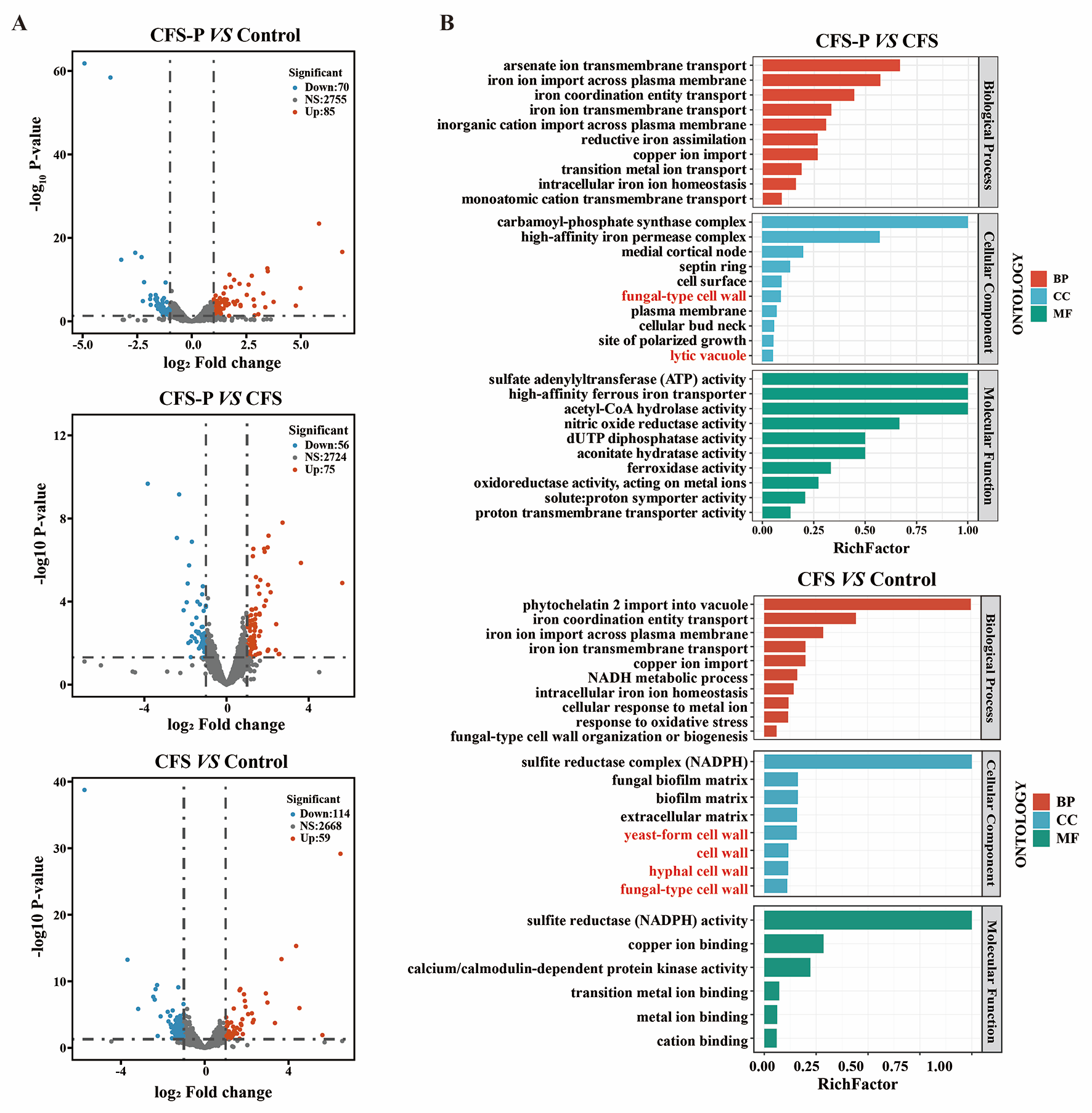


**Figure. S19.** Transcriptome sequencing analysis results. (A) Volcano plot of DEGs in *C.albicans* comparing with CFS-P, CFS, and control. (B) GO annotation analysis of DEGs in *C.albicans*.


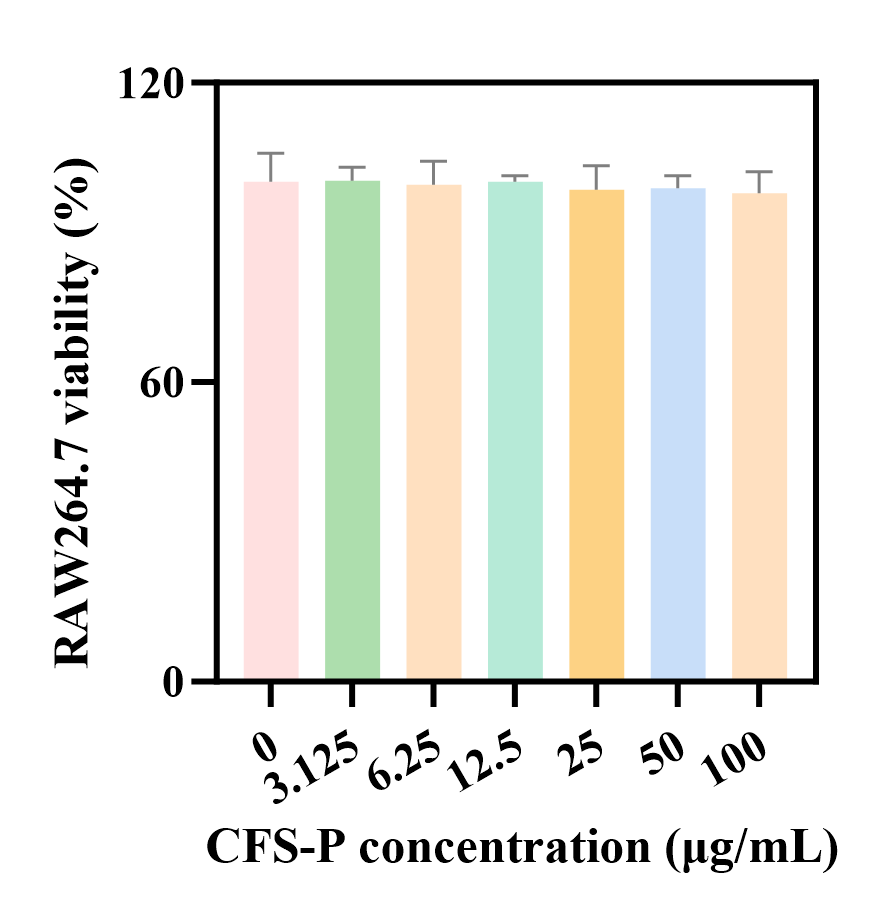


**Figure. S20.** Viability of RAW264.7 cells after treatment with different concentrations of CFS-P. (Data were presented as mean ± sd, n=6)


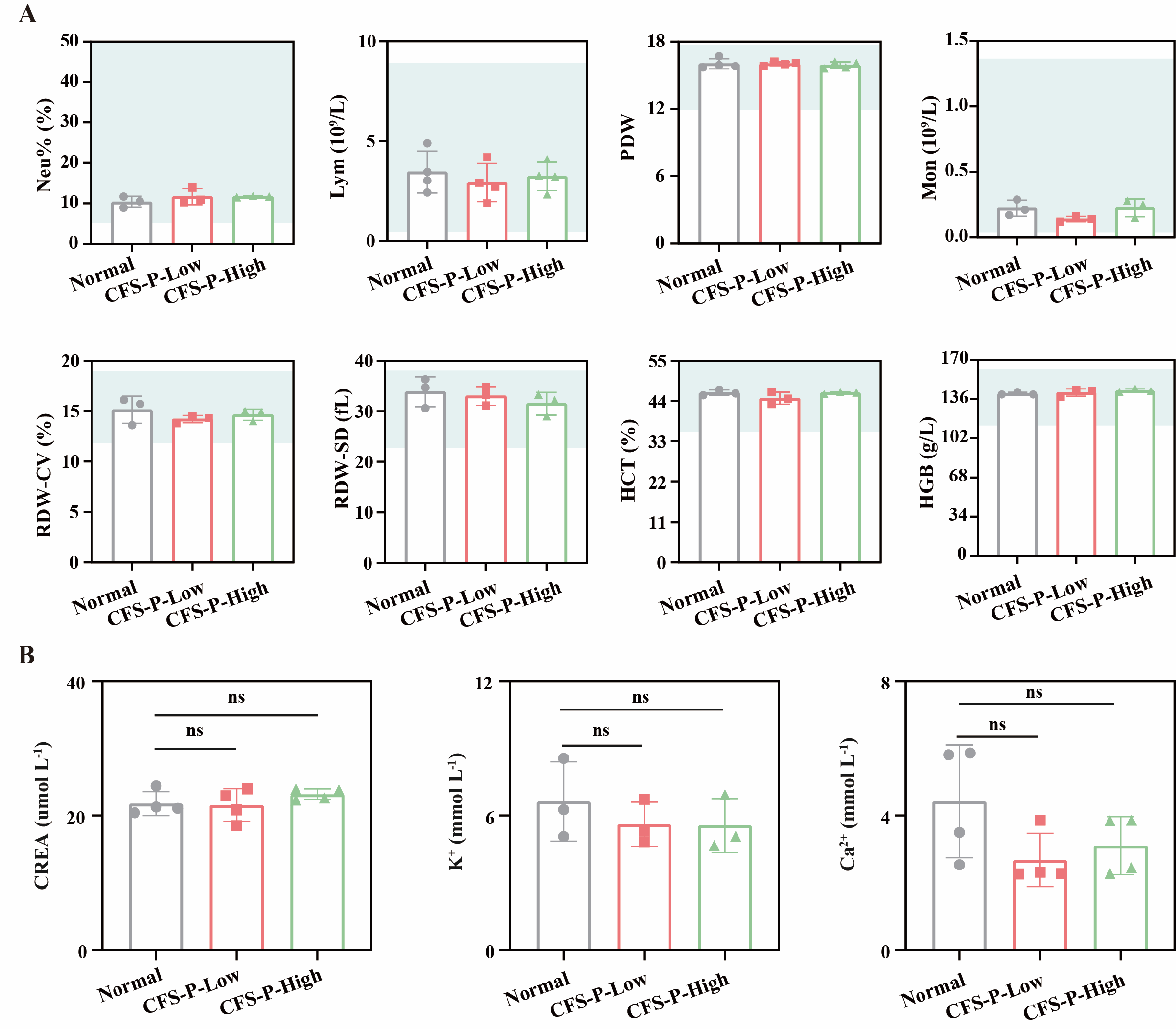


**Figure. S21.** Complete blood count (A) and serum biochemical analysis (B) of ICR mice on day 3 after intravenous injection of CFS-P.
